# Psychometric properties of the Alzheimer’s Disease Cooperative Study – Activities of Daily Living for Mild Cognitive Impairment (ADCS-MCI-ADL) scale: a post hoc analysis of the ADCS ADC-008 trial

**DOI:** 10.1186/s12877-022-03527-0

**Published:** 2023-03-06

**Authors:** Michele Potashman, Menglan Pang, Muna Tahir, Saeid Shahraz, Sascha Dichter, Robert Perneczky, Sandra Nolte

**Affiliations:** 1 Biogen, Cambridge, MA USA; 2 ICON Plc, South San Francisco, CA USA; 3 Institute for Clinical Research and Health Policy Studies, Tufts Medical Center, Boston, MA USA; 4 Biogen, Munich, Germany; 5 Department of Psychiatry and Psychotherapy, University Hospital, LMU Munich, Munich, Germany; 6 German Center for Neurodegenerative Diseases (DZNE), Munich, Germany; 7 Munich Cluster for Systems Neurology (SyNergy), Munich, Germany; 8 Ageing Epidemiology (AGE) Research Unit, School of Public Health, Imperial College, London, UK; 9 Sheffield Institute for Translational Neuroscience (SITraN), University of Sheffield, Sheffield, UK; 10 ICON Clinical Research GmbH, Munich, Germany; 11 Department of Psychosomatic Medicine, Charité – Universitätsmedizin Berlin, corporate member of Freie Universität Berlin and Humboldt-Universität Zu Berlin, Medical Clinic, Berlin, Germany

**Keywords:** Activities of daily living, Proxy-report, Functional rating scale, Cognitive rating scale, Outcome measure, Functional decline, Psychometrics, ADCS-ADL-MCI, Alzheimer's Disease, Mild Cognitive Impairment

## Abstract

**Background:**

The Alzheimer's Disease Cooperative Study – Activities of Daily Living Scale for use in Mild Cognitive Impairment (MCI), the ADCS-ADL-MCI, is an evaluation scale with information provided by an informant/caregiver to describe the functional impairment of patients with MCI. As the ADCS-ADL-MCI has yet to undergo a full psychometric evaluation, this study aimed to evaluate the measurement properties of the ADCS-ADL-MCI in subjects with amnestic MCI.

**Methods:**

Measurement properties, including item-level analysis, internal consistency reliability, test–retest reliability, construct validity (convergent/discriminant, known-groups validity), and responsiveness were evaluated using data from the ADCS ADC-008 trial, a 36-month, multicenter, placebo-controlled study in 769 subjects with amnestic MCI (defined by clinical criteria and a global clinical dementia rating, CDR, score of 0.5). Due to most subjects' mild condition at baseline and resulting low variance in scores, psychometric properties were assessed using both baseline and 36-month data.

**Results:**

Ceiling effects were not apparent at the total score level, with 3% of the cohort reaching the maximum score of 53, despite most subjects having a relatively high score at baseline (mean score = 46.0 [standard deviation = 4.8]). Item-total correlations were overall weak at baseline, most likely due to low variability in responses; however, at month 36, good item homogeneity was found. Cronbach's alpha values ranged from acceptable (0.64 at baseline) to good (0.87 at month 36), indicating overall very good internal consistency reliability. Further, moderate to good test–retest reliability was found (intraclass correlation coefficients ranging from 0.62–0.73). The analyses also largely supported convergent/discriminant validity, especially at month 36. Finally, the ADCS-ADL-MCI discriminated well between groups showing good known-groups validity, and was responsive in patients who indicated a longitudinal change in other instruments.

**Conclusions:**

This study provides a comprehensive psychometric evaluation of the ADCS-ADL-MCI. Findings suggest that the ADCS-ADL-MCI is a reliable, valid and responsive measure capable of capturing functional abilities in patients with amnestic MCI.

**Trial registration:**

ClinicalTrials.gov Identifier: NCT00000173.

**Supplementary Information:**

The online version contains supplementary material available at 10.1186/s12877-022-03527-0.

## Background

Mild cognitive impairement (MCI) is a common disorder in older populations with its prevalence increasing with age, as demonstrated by the findings that an estimated 6.7% of persons age 60–64 and 25% of persons age 80–84 are living with MCI globally [1]. While MCI disease etiology can vary, it is estimated that more than half of those with MCI have underlying disease pathology associated with Alzheimer’s disease (AD) [2]. AD is a progressive neurodegenerative disorder and is the most common cause of dementia, relfecting 60–80% of the estimated 50 million cases of dementia globally [3, 4].

The current view of AD is that the disease process and clinical manifestations are distributed along a continuum of decline, with symptoms reflecting mild cognitive impairment (MCI) occurring between "normal aging" and dementia [5]. MCI can have both amnestic and non-amnestic presentations, with symptoms of amnestic MCI occuring frequently in patients with MCI due to AD [5]. Amnestic MCI manifests [6] as objective impairments in one or more cognitive domains (typically including memory), with the potential for mild/initial impairments in select activities of daily living (ADLs) [7], typically those activities that require more cognitive complexity such as managing finances and medications. These activities known as instrumental ADLs (IADLs) are affected well before changes are noticeable in basic ADLs (BADLs), such as personal hygiene, dressing, and feeding [8]. Notably, while there may be detectable impacts on IADLs in patients with MCI, impairments in BADLs are absent, as this would qualify the patient to be classified as having AD dementia [7, 9, 10].

Given the array of cognitive and functional impairments observed across the AD continuum, selecting suitable clinical outcome assessment (COA) instruments for measuring the effects of treatments in clinical trials with early symptomatic patients requires careful consideration. As AD patients may have a reduced ability to understand the impact of their disease on daily functioning, even early in the disease course [11], caregiver reports are typically incorporated to assess AD patients' cognitive and physical functioning in areas such as ADLs and quality of life.

The Alzheimer's Disease Cooperative Study – Activities of Daily Living Scale for use in Mild Cognitive Impairment (ADCS-ADL-MCI) is a functional evaluation scale with the information provided by an informant/caregiver to describe the performance of patients in several ADLs. This measure is commonly used in clinical trials and clinical practice to measure the functional evolution of patients with MCI [12–15]. However, while there is evidence of concurrent validity [8, 16] and known-groups validity, there is limited evidence on other psychometric properties of the instrument, such as floor/ceiling effects, reliability, or responsiveness. When considering the parent measure for use in patients with AD dementia (ADCS-ADL), there is evidence on convergent/discriminant validity [17], known-groups validity [17, 18], and internal consistency reliability [18] for the mild AD dementia population.

In light of the evidence gaps regarding the measurement properties of the ADCS-ADL-MCI, the present study focused on the assessment of the psychometric properties of this instrument in subjects with amnestic MCI, including item-level analyses, internal consistency reliability, and homogeneity, test–retest reliability, construct validity (convergent and discriminant validity, known-groups validity), and responsiveness/sensitivity to change, using data from a large randomised controlled trial.

## Methods

### Data source and analysis population

The psychometric evaluation of the ADCS-ADL-MCI was undertaken using data from the ADCS ADC-008 trial, a 36-month, multicenter, randomized, double-blind, parallel-group, placebo-controlled study in subjects with amnestic MCI [19]. All subjects were aged 55–91 years (inclusive) and met criteria for amnestic MCI of a presumably degenerative nature (insidious onset, gradual progression) defined as: 1) subjective memory complaint corroborated by an informant; 2) general cognition and functional ability sufficiently preserved so that a diagnosis of AD dementia or non-AD dementia could not be made; 3) abnormal memory function defined as scoring below the education-adjusted normative cutoff value on one paragraph from the Wechsler Memory Scale-Revised Logical Memory II subtest, 4) a global Clinical Dementia Rating score of 0.5, and 5) a Mini-Mental State Examination (MMSE) score ≥ 24 [20]. Clinical diagnosis was assigned absent biomarker evidence of disease pathology. Eligible subjects who completed the screening visit and gave informed consent were randomly assigned to one of the following three treatment groups in a double-blind fashion: Placebo plus a multivitamin daily (*n* = 259); Vitamin E (2,000 IU) plus a multivitamin daily (*n* = 257); or donepezil (10 mg) plus a multivitamin daily (*n* = 253). The primary outcome was the development of possible or probable AD dementia.

The science of evaluating an instrument’s measurement properties does not consider the biological mechanism of the underlying disease process and is agnostic as to the reasons why patients are in the evaluated disease state. As such, disease experiences as viewed through clinical measures and patient-reported outcomes resulting from treatment are considered equally usable to non-treated natural history patients (i.e., the instrument is applicable across the disease continuum). The psychometric evaluation of the ADCS-ADL-MCI was thus performed on all randomized subjects in the ADC-008 dataset for whom the ADCS-ADL-MCI was measured (*N* = 769). Further details of this trial are reported elsewhere [19]. All methods were carried out in accordance with relevant guidelines and regulations under IRB #981135.

### ADCS-ADL-MCI

The ADCS-ADL-MCI is a functional evaluation scale that assesses the ability of patients to perform ADLs (with recall "In the past 4 weeks") through a structured questionnaire administered to the informant/carer by a physician or qualified rater [21]. The ADCS-ADL-MCI was derived from its parent measure, the ADCS-ADL [12], and adapted to be suitable for MCI patients. Two forms of the ADCS-ADL-MCI are the 18-item and the 24-item version. While the 24-item version was administered as part of the ADC-008 trial, the 18-item version is explored in the present study due to practical considerations (i.e., its use in current clinical trials such as the phase 3 aducanumab trials EMERGE [NCT 02484547] and ENGAGE [NCT 02477800]). Items in the measure predominantly include IADLs, such as balancing a checkbook, navigating outside the home, shopping, using household appliances, or finding personal belongings. Physically getting dressed and the ability to be left alone are also being assessed. The 18-item ADCS-ADL-MCI is scored from 0–53 based on the subject's degree of independence in performing specific tasks (i.e., independent, partially independent, fully dependent). Except for item 3 (i.e., 'Regarding physically getting dressed, which best describes his/her usual performance in the past 4 weeks?', with response options ranging from '0 = Someone else dressed him/her' to '4 = Dressed completely without supervision or physical help'), all other items follow a gating response format [22]. That is, the first step of the item asks about whether the patient performed the specific task – and in case of endorsement – the second step usually follows the response format ranging from '1 = With physical help' to '3 = Without supervision or help' or a similar wording probing the details and manner of performing the specific task. Some items include four to five response options that describe how the IADL was performed (rather than three response options as in the more standardized response format for items 1, 2, 4, 5, and 7). Further, the second step of some items includes subquestions that are more descriptive regarding a particular activity (such as watching television), including only 'yes' and 'no' response options. Lower scores on the measure denote more substantial impairment [12, 16]. In this study, the ADCS-ADL-MCI was assessed at baseline and months 6, 12, 18, 24 and 36 for patients with MCI. Per the ADC-008 trial design, patients who progressed to mild AD dementia were moved into an open-label study with donepezil, and where the ADCS-ADL-MCI was not included in that study design. Consequently, these patients are no longer included in the analysis after having progressed to mild AD dementia and, therefore, this study does not provide any information on the measurement properties of the ADCS-ADL-MCI as applicable to patients with mild AD dementia.

### Other clinical outcome assessment (COA) instruments

To be able to assess the range of psychometric properties of the ADCS-ADL-MCI as detailed further below, several additional COA questionnaires were used that had been completed by patients, their caregivers, or clinicians at various time points. These questionnaires included the Global Deterioration Scale (GDS), the Informant Beck Depression Inventory (BDI), the NYU Delayed Paragraph Recall Test, and the Symbol Digit Modalities Test. Further, the Clinical Dementia Rating – Sum of Boxes (CDR-SB), the 11-item Alzheimer's Disease Assessment Scale – Cognitive Subscale (ADAS-Cog), and the Mini-Mental-State Examination (MMSE) were collected at screening and months 6, 12, 18, 24, and 36, while the Quality of Life – Alzheimer's Disease (QoL-AD) was assessed at baseline and months 6, 12, 24, and 36. Finally, the Mild Cognitive Impairment – Clinician Global Impression of Change (MCI-CGIC) was assessed at months 6 and 12, while *APOE* ε4 allele carrier status was assessed at screening only.

### General analysis considerations

Analyses were performed in accordance with a pre-specified statistical analysis plan (SAP). For the majority of the psychometric properties tested, screening/baseline and month 36 data were primarily used in the analyses; test–retest leveraged data from screening/baseline and month 6; responsiveness to change leveraged data from screening/baseline and month 36.

The initial focus of the psychometric tests was baseline data, except for those tests that typically include follow-up data, such as test–retest or responsiveness analyses. However, initial analyses showed that the MCI population had limited functional impairments at baseline as reflected by low variability of the data and various ADCS-ADL-MCI items exhibiting ceiling effects. Further, some of the reference COA instruments were assessed at screening only (i.e., CDR-SB, ADAS-Cog, and MMSE), while the ADCS-ADL-MCI was assessed at baseline. Per protocol, the baseline was assessed between three and five weeks after the screening visit. While it is not expected that change occurred between these two assessments, ideally, data are evaluated on the same day to ensure that analyses, such as correlation analyses, are not affected by potential alternative effects such as different assessment time points. Therefore, for these two reasons the 36-month data were additionally included for all psychometric tests where applicable (e.g., due to increased variability of scores observed (due to progression over the 36 months in the study) and the fact that all COAs were assessed at the same time point). While evaluation of only placebo patients at month 36 was considered, the richness and real-world relevance of information contained in the full population was deemed more informative. Indeed, a heterogeneous cohort of patients was envisioned, with varying degrees of disease progression, which was considered helpful to evaluate the measurement properties. It is noted that patients at the 36-month timepoint do not include those who had progressed to dementia, and thus the sample only reflects those with slower disease progression.

### Item-level analyses

Item-level analyses (i.e., item variability or frequency of endorsement) were evaluated using baseline and 36-month data, and items were further explored by sex, given the potential for certain items to differ by sex based on content. The item-level analyses included measures of central tendency (to assess the distribution of total scores), use of response categories for each item (i.e., frequency and percentage of patients in each response category), and an assessment of floor and ceiling effects at the item-level (using the sub questions, rather than the gated questions) and total score-level [23].

### Reliability

Internal consistency reliability (i.e., the extent to which individual items within an instrument correlate with each other to form a multi-item scale) was assessed at baseline and month 36 using item-total correlations, with a target significant correlation > 0.30 indicating good homogeneity [24, 25], and using the Cronbach's alpha coefficient, with a target value of 0.7 indicating good internal consistency [25, 26].

Test–retest reliability (i.e., the extent to which a measure yields consistent scores within the same patient each time it is administered) was assessed between baseline and month 6 (given the study design) among stable patients. Stability was defined in two ways: patients with no change in each of a) MCI-CGIC, GDS, and CDR-SB; or alternatively b) MCI-CGIC, GDS, and MMSE. As test–retest reliability is typically assessed across shorter time periods where there can be a strong rationale for disease stability (e.g., a few days to two weeks between measurement timepoints), we sought assurance that the disease was stable. Therefore a three-component definition was employed using 1) a disease-specific assessment of global disease severity (MCI-CGIC), 2) a non-disease-specific global measure of disease severity (GDS) and 3) a detailed measure of disease severity (CDR-SB or MMSE). We examined both the CDR-SB and MMSE as we proposed the CDR-SB to be the more accurate measure of disease severity and the MMSE to be a measure with more widespread use (but higher variability in the MCI population). Mean differences and paired t-tests were calculated to compare ADCS-ADL-MCI total scores (and the statistical significance of any change) between the two assessment visits. To assess test–retest reliability, the intraclass correlation coefficients (ICC) was computed where ≥ 0.7 indicates good reproducibility [27, 28].

### Validity

Convergent/discriminant validity (i.e., the degree to which a measure is associated with other measures/variables conceptually or based on the expected relationship with the chosen variable(s)) was assessed using screening/baseline data by examining correlations of the ADCS-ADL-MCI with various COA measures included in the trial. Specifically, the ADCS-ADL-MCI was anticipated to be at least moderately correlated (> 0.30) [29, 30] with the CDR combined functional domains and the GDS, and less correlated with the CDR combined cognitive domains, the ADAS-Cog, the MMSE, as the ADCS-ADL-MCI items cover primarily functional concepts. However, it is also known that in this early stage of impairment, the division between cognitive tasks and functional abilities may not be clearly demarcated, so rather than setting "thresholds" for convergent and divergent validity assessment, a descriptive "matrix" was pursued. Similarly, the BDI and the Qol-AD-Informant were anticipated to demonstrate convergent validity as well but to a lesser extent than the former instruments, as depression and quality of life aspects might be more causally related to ADLs rather than be measuring similar constructs. Lower correlations were anticipated between the ADCS-ADL-MCI, and the NYU delayed paragraph recall test, and the Symbol Digit Modalities test, as these instruments measure different cognitive constructs focused on verbal memory and information processing speed/efficiency. Of note, due to the aforementioned low variability in scores at baseline, and the fact that some of the included reference measures had been collected at screening rather than at baseline like the ADCS-ADL-MCI (i.e., the CDR-SB, ADAS-Cog, and MMSE), convergent/discriminant validity was also assessed using 36-month data. Sensitivity analyses were also conducted to explore convergent validity among only patients who had both baseline and 36-month data. Given that the assumption of linearity may not be achieved with the various measures used in the study (including the ADCS-ADL), a more conservative statistical approach was taken using Spearman’s correlations to assess this psychometric property.

Known-groups validity (i.e., the ability of an instrument to distinguish between different populations when a difference between them is expected) was examined at baseline (and at month 36 for consistency with the other psychometric tests) to determine whether the ADCS-ADL-MCI could distinguish between patients by disease severity within the MCI spectrum. Thereby, disease severity was defined in several ways: 1) *APOE* ε4 allele carrier status (carrier/non-carrier); 2) GDS categories (no/very mild, mild and moderate cognitive decline); 3) MMSE categories (< 27 vs. ≥ 27); 4) MMSE quintiles; 5) BDI categories (normal and abnormal, including mild mood disturbance, borderline clinical depression, moderate depression, severe depression, extreme depression); 6) BDI quintiles; 7) CDR-SB categories (< 2.5 vs. ≥ 2.5); and 8) CDR-SB quintiles. Analysis of variance (ANOVA) models were used to assess differences in ADCS-ADL-MCI scores by severity groups, respectively.

### Responsiveness

Finally, responsiveness (i.e., the ability of an instrument to measure any degree of change when a known change in the concept of interest has occurred) was assessed using specific anchor measures to define groups of patients showing a change from baseline to month 36 based on the anchor measures. Specifically, patients were classified as 'improved,' 'no change,' and 'worsened' based on their change scores on 1) GDS; 2) CDR-SB; 3) global CDR, and 4) CDR-SB functional domain score. For example, patients who had a ≥ -1 point decrease on the GDS between baseline and month 36 were classified as 'improved.' Patients who had a ≥ 1 point increase on the GDS between baseline and month 36 were classified as 'worsened.' Stable patients were defined as those who did not experience any change in their condition according to the GDS between baseline and month 36 (i.e., 0 point change on the GDS).

Mean change between baseline and month 36 and effect size (ES) (i.e., mean change divided by baseline standard deviation) were calculated for the ADCS-ADL-MCI total score within each change category, and effect sizes were compared [30]. ANOVA was used to determine whether the difference was statistically significant between the groups and whether there was any linear trend in change scores.

### Factor analysis

To explore the dimensionality of the ADCS-ADL-MCI, both exploratory and confirmatory factor models were run. However, the models generally did not converge which is likely due to the low variance at baseline. In addition, the gating response format adds further complexity to running such models. Therefore, as results from the factor analyses were inconclusive, these are not reported in the results section.

SAS version 9.4 (SAS Institute Inc, Cary, NC) was used for all statistical analyses detailed above. In all of the analyses, *p* < 0.05 was considered significant.

## Results

### Sample description

The baseline characteristics of subjects with MCI included in the ADC-008 sample are presented in Table 1. Patients in the ADC-008 trial were predominantly white (92%), while just over half of the sample were male (54%) and/or *APOE* ε4 allele carriers (55%). The average age was 73 years (SD = 7.3). The mean number of years of education was 15 years (SD = 3.1), while the mean number of years since symptom onset was three years (SD = 2.6).

**Table 1:** Baseline characteristics of the ADC-008 sample

Characteristic	Overall	Male	Female
N (%)	769 (100)	417 (54.23)	352 (45.77)
Age, mean (SD)	72.92 (7.31)	72.81 (7.19)	73.05 (7.45)
Sex (Male), n (%)	417 (54.23)	-	-
*APOE* ε4 allele carrier, n (%)	424 (55.14)	221 (53.00)	203 (57.67)
Years since disease onset, mean (SD)	3.02 (2.62)	3.18 (2.57)	2.83 (2.68)
Years of education, mean (SD)	14.64 (3.08)	15.12 (3.21)	14.07 (2.82)
Ethnicity, n (%)			
American Indian or Alaskan Native	3 (0.39)	0 (0)	3 (0.85)
Asian or Pacific Islander	7 (0.91)	2 (0.48)	5 (1.42)
Black; not of Hispanic Origin	18 (2.34)	7 (1.68)	11 (3.13)
Hispanic	30 (3.90)	12 (2.88)	18 (5.11)
White; not of Hispanic Origin	708 (92.07)	394 (94.48)	314 (89.20)
Other or Unknown	3 (0.39)	2 (0.48)	1 (0.28)

*SD* Standard Deviation

At baseline, the mean ADCS-ADL-MCI score was 46 (SD = 4.8), and most patients (57%) had mild cognitive decline defined by categories of GDS. The mean scores for the MMSE, CDR-SB, and ADAS-Cog at screening were 27.27 (SD = 1.9), 1.82 (SD = 0.8), and 11.28 (SD = 4.4), respectively. Scores on the other reference measures assessed in the trial are presented in Table 2, including baseline and 36-month data. Significant differences in scores were observed between baseline and month 36 scores, such that disease severity appeared to worsen at month 36 compared to baseline. Further, at month 36, 54.75% (*N* = 265) of the cohort were APOE4 carriers.

**Table 2:** Scores on reference measures included in the ADC-008 trial at baseline and month 36

Instrument	Baseline (*n* = 769)	Month 36 (*n* = 484)
ADCS-ADL-MCI ^a^¥		
Mean (SD)	45.95 (4.77)	40.63 (10.97)
Median (IQR)	47 (44—49.5)	45 (35—49)
Min, Max	18, 53	3, 53
Missing (n)	1	7
Global Deterioration Scale, n (%)¥		
No cognitive decline	4 (0.52)	9 (1.86%)
Very mild cognitive decline	289 (37.58)	134 (27.69%)
Mild cognitive decline	436 (56.70)	165 (34.09%)
Moderate cognitive decline	40 (5.20)	91 (18.80%)
Moderately severe cognitive decline	0 (0)	68 (14.05%)
Severe cognitive decline	0 (0)	11 (2.27%)
Missing (n)	0	6
MMSE, mean (SD) ¥	27.27 (1.85)	25.28 (4.79)
Missing (n)	0	4
CDR-SB, mean (SD) ¥	1.82 (0.79)	3.17 (2.81)
Missing (n)	0	11
ADAS-Cog, mean (SD) ^a^	11.28 (4.38)	14.05 (8.73)
Missing (n)	4	11
BDI, mean (SD)	6.85 (5.18)	8.55 (6.30)
Missing (n)	11	15
Qol-AD Informant Score, mean (SD) ¥	39.28 (5.49)	37.07 (6.46)
Missing (n)	22	24
NYU delayed paragraph recall test, mean (SD) ¥	3.54 (2.80)	2.58 (2.66)
Missing (n)	1	9
Symbol digit modalities test, mean (SD) ¥	31.58 (10.73)	32.19 (13.22)
Missing (n)	2	17

^a^ Small inconsistencies in scores compared to those reported by Petersen et. al (2005) [19]

¥ Statistically significant difference in scores between baseline and month 36 (*p*-value < 0.05) was detected, based on data available at both time points

*SD* Standard Deviation, *IQR* Interquartile Range

### Item-level analyses

Item-level analyses and the distribution of ADCS-ADL-MCI total scores for the full sample and by sex at baseline and month 36 are shown in Supplemental Tables 1 and 2, respectively (see Additional file 1).

At baseline, about two-thirds of individual items exhibited ceiling effects, i.e., a generally high level of ability of most patients regarding ADLs. Specifically, items 1, 2, 3, 10, 11, 12a-c, 13a, 14a, 15a-b, 16a-c, 17a, and 18a had strong ceiling effects, with over 80% of subjects being assessed as carrying out the respective activity "without supervision". Likewise, at month 36, most of these ceiling effects at the item level remained but to a lesser extent (i.e., no longer evident for items 1, 10 and 11). Further, some differences were observed by sex for specific items, such that a lower proportion of males were endorsed in the first step of the gating question. This finding applied to items assessing cleaning or loading laundry; for example, 67% of males versus 88% of females were evaluated as having cleaned (item 4), and 45% of males versus 96% of females were assessed as having loaded laundry (item 7) in the past four weeks. Similar differences by sex were observed at month 36.

At the total ADCS-ADL-MCI score level, the observed ceiling effects at baseline and month 36 were no longer apparent. With a maximum possible total score of 53, less than 3% of patients reached the top score, while less than 15% reached one of the top three scores at baseline (i.e., reached a score ≥ 51). The overall distribution of scores at baseline was mean (standard deviation, SD) = 45.95 (4.77) and median (interquartile range, IQR) = 47 (44–49.5). Similarly, less than 1% of patients reached the top score, and less than 13% reached one of the top three scores at month 36 (i.e., reached a score ≥ 51). The overall distribution of scores at month 36 was mean (SD) = 40.63 (10.97) and median (IQR) = 45 (35, 49).

### Reliability

At baseline, most items in the ADCS-ADL-MCI did not correlate well with the total score of the remaining items (i.e., did not meet the pre-specified threshold of 0.30). Four of the 18 ADCS-ADL-MCI items had correlations of > 0.30 with the total score, generally suggesting low item homogeneity (items 6, 9, 13 and 14). In contrast, item-total correlations were higher at month 36, with all but one item (item 7) having correlations between 0.30 and 0.70. Acceptable internal consistency was demonstrated at baseline with an overall Cronbach's alpha value of 0.64. Cronbach's alphas with each item omitted were largely similar, ranging between 0.58 and 0.64. Similar to the item-total correlations, Cronbach's alphas were higher at month 36 (overall Cronbach's alpha = 0.87), indicating good internal consistency. Cronbach's alphas with each item omitted were similar, ranging between 0.85 and 0.87 (Table 3).

**Table 3:** Item-total correlations and Cronbach's alphas for the ADCS-ADL-MCI at baseline and month 36

ADCS-ADL-MCI Item	Item-total correlations Spearman Correlations (*p*-value)^a^		Cronbach’s alpha^b^	
	Baseline (*n* = 768)	Month 36 (*n* = 477)	Baseline (*n* = 768)	Month 36 (*n* = 477)
Item 1	0.14 (0.0002)	0.47 (< .0001)	0.633/0.616	0.863/0.891
Item 2	0.13 (0.0005)	0.44 (< .0001)	0.631/0.614	0.864/0.889
Item 3	0.07 (0.0473)	0.37 (< .0001)	0.636/0.628	0.867/0.892
Item 4	0.08 (0.0418)	0.42 (< .0001)	0.635/0.623	0.867/0.893
Item 5	0.14 (0.0027)	0.42 (< .0001)	0.630/0.612	0.869/0.893
Item 6	0.32 (< .0001)	0.49 (< .0001)	0.611/0.605	0.862/0.892
Item 7	0.14 (0.0011)	0.28 (< .0001)	0.634/0.617	0.870/0.896
Item 8	0.24 (< .0001)	0.64 (< .0001)	0.618/0.609	0.854/0.885
Item 9	0.32 (< .0001)	0.67 (< .0001)	0.591/0.589	0.853/0.884
Item 10	0.21 (< .0001)	0.50 (< .0001)	0.641/0.621	0.866/0.893
Item 11	0.22 (< .0001)	0.60 (< .0001)	0.617/0.604	0.856/0.886
Item 12	0.24 (< .0001)	0.52 (< .0001)	0.601/0.596	0.862/0.891
Item 13	0.34 (< .0001)	0.56 (< .0001)	0.592/0.600	0.861/0.892
Item 14	0.39 (< .0001)	0.62 (< .0001)	0.582/0.594	0.856/0.889
Item 15	0.26 (< .0001)	0.55 (< .0001)	0.617/0.592	0.860/0.887
Item 16	0.17 (< .0001)	0.46 (< .0001)	0.626/0.617	0.864/0.890
Item 17	0.13 (0.0004)	0.46 (< .0001)	0.635/0.630	0.864/0.890
Item 18	0.12 (0.0007)	0.37 (< .0001)	0.632/0.618	0.867/0.893
Total Score: Cronbach’s alpha	-	-	0.635/0.624	0.869/0.896

^a^ADCS-ADL-MCI total score with each item deleted

^b^Cronbach's alpha with each item deleted (raw / standardized)

Test–retest reliability among stable patients based on the MCI-CGIC, GDS and CDR-SB (*n* = 108 reporting no change on all three measures) was supported with an ICC value of 0.73 and a minimal, non-significant change between baseline and month 6 (0.13 points, *p* = 0.67; Table 4). While there was also a minimal, non-significant decrease in ADCS-ADL-MCI scores between baseline and month 6 when the MCI-CGIC, GDS, and MMSE were used to define stable patients (-0.2 points, *p* = 0.68), the ICC value for this group of patients was lower at 0.62, indicating moderate test–retest reliability (*n* = 69 reporting no change on all three measures).

**Table 4:** Test–retest reliability: ADCS-ADL-MCI mean change scores and intraclass correlation coefficients between baseline and month 6

Definition of Stable Patients ^a^	N (%)	Baseline Mean (SD) ADCS-ADL-MCI Total Score	Month 6 Mean (SD) ADCS-ADL-MCI Total Score	Mean Change (SD) in ADCS-ADL-MCI Total Score	t-test (*p*-value)	Intraclass Correlation Coefficient (95% Confidence Interval)
MCI-CGIC, GDS and CDR-SB	108 (16.49)	46.76 (4.35)	46.89 (4.21)	0.13 (3.19)	0.673	0.73 (0.62, 0.80)
MCI-CGIC, GDS and MMSE	69 (10.53)	46.90 (4.15)	46.70 (4.95)	-0.20 (4.02)	0.676	0.62 (0.45, 0.74)

*N* = 655 patients had an ADCS-ADL-MCI total score measured at both baseline and month 6

^a^ Stable patients defined as those who experienced "no change" on the MCI-CGIC at month 6 and those with a 0-point change between baseline and month 6 on the GDS and CDR-SB or on the GDS and MMSE

### Validity

Correlations between ADCS-ADL-MCI total scores and other relevant COA measures are presented in Table 5. At baseline, a moderate correlation (r ≥ 0.30) was observed between the ADCS-ADL-MCI total score and the CDR-SB total score, indicating convergent validity as expected. The correlations between the ADCS-ADL-MCI total scores and other COA instruments were all statistically significant but relatively weak (< 0.30), with no apparent differences between those instruments expected to show convergent validity as opposed to those expected to show discriminant validity. In contrast, at month 36, the results of the correlation analyses between the ADCS-ADL-MCI and the other instruments showed a substantially different and much clearer picture, now largely confirming the a priori defined expectations. Specifically, the ADCS-ADL-MCI demonstrated strong correlations with the CDR-SB total score and each domain score, with correlations ranging between -0.77 for the total score and -0.52 for the personal care domain. Correlations with the ADAS-Cog, MMSE, and GDS were also large, ranging from │0.64│ to │0.73│. Moderate correlations were observed between the ADCS-ADL-MCI and the BDI and Qol-AD-Informant total score. As opposed to our a priori expectations, both the NYU delayed paragraph recall test and Symbol Digit Modalities test also showed moderate correlations with the ADCS-ADL-MCI. Sensitivity analyses suggested almost identical findings when data at baseline were restricted to only those who also had 36-month data (data not shown).

**Table 5:** Convergent /discriminant validity: Spearman correlations between ADCS-ADL-MCI scores and other ADC-008 trial instruments at baseline/month 36

Instrument	Baseline	Month 36
	r (*p*-value)	
CDR-SB Total Score	-0.33 (< .0001)	-0.77 (< .0001)
CDR Cognitive Domain Score	-0.27 (< .0001)	-0.74 (< .0001)
CDR Functional Domain Score	-0.27 (< .0001)	-0.76 (< .0001)
CDR Home and Hobbies Domain	-0.19 (< .0001)	-0.71 (< .0001)
CDR Community Affairs Domain	-0.26 (< .0001)	-0.74 (< .0001)
CDR Personal Care Domain	-0.10 ( 0.0055)	-0.52 (< .0001)
CDR Memory Domain	-0.17 (< .0001)	-0.72 (< .0001)
CDR Judgment and Problem Solving Domain	-0.11 ( 0.0015)	-0.65 (< .0001)
CDR Orientation Domain	-0.26 (< .0001)	-0.69 (< .0001)
ADAS-Cog Total Score	-0.24 (< .0001)	-0.65 (< .0001)
MMSE Total Score	0.2 (< .0001)	0.64 (< .0001)
GDS	-0.19 (< .0001)	-0.73 (< .0001)
BDI	-0.23 (< .0001)	-0.51 (< .0001)
Qol-AD-Informant Total Score	0.19 (< .0001)	0.46 (< .0001)
NYU Delayed Paragraph Recall Test	0.25 (< .0001)	0.56 (< .0001)
Symbol Digit Modalities Test	0.24 (< .0001)	0.52 (< .0001)

In view of known-groups validity, the ADCS-ADL-MCI was able to differentiate patients by disease severity (*p* < 0.01) based on the GDS, MMSE, BDI, and CDR-SB at baseline and month 36 (Table 6). That is, ADCS-ADL-MCI total scores scales were lower (indicating more severe disease) with higher GDS, BDI, and CDR-SB scores and lower with lower MMSE scores (indicating higher disease severity). The pattern of ADCS-ADL-MCI total scores was generally monotonous, i.e., worse/lower ADCS-ADL-MCI scores with increasing severity according to the other instruments, providing strong support for known-groups validity. While no group differences in ADCS-ADL-MCI total scores were observed by *APOE* ε4 allele carrier status at baseline, significant differences were demonstrated at month 36 (i.e., lower scores observed among the carriers; *p* < 0.001).

**Table 6:** Known-groups validity: Differences in ADCS-ADL-MCI scores between severity groups at baseline and month 36

Group Definition	Baseline			Month 36		
	N	Mean (SD)	ANOVA/t-test (*p*-value)	N	Mean (SD)	ANOVA/t-test (*p*-value)
*APOE* ε4 allele carrierStatus						
Carrier	423	46.04 (4.41)	0.5443	262	38.02 (11.44)	< 0.001
Non-Carrier	345	45.83 (5.19)		215	43.80 (9.45)	
GDS Categories						
No/very mild cognitive decline	293	46.76 (4.89)	< 0.001	141	48.16 (3.54)	< 0.001
Mild cognitive decline	435	45.67 (4.59)		163	44.56 (6.12)	
Moderate cognitive decline	40	43.00 (4.52)		170	30.65 (11.29)	
MMSE Categories						
≥ 27	505	46.45 (4.57)	< 0.001	250	46.24 (5.65)	< 0.001
< 27	263	44.99 (5.01)		227	34.45 (12.04)	
MMSE Quintiles						
Quintile 1 (> 29)	93	47.59 (3.62)	< 0.001	73	48.4 (3.64)	< 0.001
Quintile 2 (> 28 and ≤ 29)	143	47.01 (4.20)		73	46.56 (4.65)	
Quintile 3 (> 27 and ≤ 28)	135	46.21 (4.11)		54	44.72 (7.38)	
Quintile 4 (> 25 and ≤ 27)	240	45.36 (5.13)		91	43.64 (6.68)	
Quintile 5 (≤ 25)	157	44.69 (5.34)		186	32.59 (12.1)	
BDI Categories						
Normal (0–10)	587	46.34 (4.41)	< 0.001	309	43.5 (9.07)	< 0.001
Abnormal (> 10)	171	44.64 (5.56)		158	34.82 (12.25)	
BDI Quintiles						
Quintile 1 (≤ 2)	159	47.66 (3.94)	< 0.001	84	47.88 (5.36)	< 0.001
Quintile 2 (> 2 and ≤ 5)	205	46.42 (4.14)		91	44.21 (7.62)	
Quintile 3 (> 5 and ≤ 7)	99	45.55 (4.34)		59	43.14 (8.73)	
Quintile 4 (> 7 & ≤ 11)	165	45.42 (4.94)		95	38.51 (11.12)	
Quintile 5 (> 11)	130	44.14 (5.73)		138	34.02 (12.3)	
CDR-SB Categories						
< 2.5	545	46.93 (4.11)	< 0.001	234	47.57 (4.17)	< 0.001
≥ 2.5	223	43.55 (5.40)		236	33.85 (11.20)	
CDR-SB Quintiles						
Quintile 1 (≤ 1)	197	47.77 (3.82)	< 0.001	148	48.09 (3.78)	< 0.001
Quintile 2 (= 1.5)	191	46.50 (4.07)		47	47.74 (3.71)	
Quintile 3 (= 2)	157	46.41 (4.36)		39	45.38 (5.36)	
Quintile 4 (= 2.5)	139	44.25 (5.02)		34	43.21 (6.47)	
Quintile 5 (> 2.5)	84	42.39 (5.82)		202	32.28 (11.07)	

### Responsiveness

Changes in ADCS-ADL-MCI scores based on the GDS, CDR-SB, global CDR score, and CDR functional domain score groups were statistically significant (*p* < 0.001; Table 7). Specifically, the worsening category of all four instruments was associated with worsening, i.e., decreases, in ADCS-ADL-MCI scores, with large effect sizes (ES > 2.0) observed for the worsening category across the groups. In contrast, negligible to moderate ES were observed for the 'improved' and 'no change' categories on all reference measures. The 'improved' group based on CDR-SB and global CDR score, respectively, thereby showed increases/improvement in ADCS-ADL-MCI scores, whilst the 'improved' group according to CDR functional domain showed a marginal decrease in ADCS-ADL-MCI scores. The only measure where the trend was inconsistent was when the GDS was used as an anchor measure.

**Table 7:** Responsiveness: Change in ADCS-ADL-MCI scores by change in other trial measures (baseline to month 36)

ADCS-ADL-MCI Total Score	N	Mean Change (SD)^a^	Mean Change (95% Confidence Interval)^a^	Median Change (IQR)	Effect Size^b^	ANOVA/t-test^c^ (*p*-value)	ANOVA p for linear trend
Change in GDS							
Improvement	56	-1.71 (5.79)	-1.71 (-3.23, -0.20)	-1 (-3, 1)	-0.51	< 0.001	< 0.001
No change	218	-0.54 (5.28)	-0.54 (-1.24, 0.16)	0 (-3, 2)	-0.13		
Worsening	200	-12.36 (10.61)	-12.36 (-13.83, -10.89)	-11 (-18, -5)	-2.75		
Change in CDR-SB							
Improvement	129	0.40 (3.73)	0.40 (-0.24, 1.05)	0 (-1, 2)	0.10	< 0.001	< 0.001
No change	69	-0.77 (4.77)	-0.77 (-1.89, 0.36)	-1 (-3, 2)	-0.21		
Worsening	272	-9.69 (10.66)	-9.69 (-10.96, -8.43)	-8 (-15, -2)	-2.16		
Change in Global CDR Score							
Improvement	21	0.57 (3.14)	0.57 (-0.77, 1.91)	1 (-1, 3)	0.13	< 0.001	< 0.001
No change	307	-1.50 (5.40)	-1.50 (-2.11, -0.90)	-1 (-4, 2)	-0.39		
Worsening	142	-15.41 (10.73)	-15.41 (-17.17, -13.64)	-13 (-22, -8)	-3.21		
Change in CDR Functional Domain Score							
Improvement	89	-0.12 (5.23)	-0.12 (-1.21, 0.96)	1 (-2, 3)	-0.03	< 0.001	< 0.001
No change	151	-0.75 (4.53)	-0.75 (-1.48, -0.03)	0 (-3, 2)	-0.20		
Worsening	231	-10.97 (10.81)	-10.97 (-12.37, -9.58)	-9 (-17, -2)	-2.50		

*SD* Standard Deviation, *ANOVA* Analysis of Variance,* IQR* Interquartile Range

^a^ Mean change between baseline and month 36

^b^ Effect size = mean change/Baseline SD

^c^ Parametric *P*-value for between-group comparisons: ANOVA/t-test for continuous variables

## Discussion

The aim of this study was the evaluation of the psychometric properties of the ADCS-ADL-MCI, a functional evaluation scale assessing aMCI patients' ability to perform ADLs as reported by an informant/carer. This psychometric evaluation included aspects of reliability, validity, and responsiveness examined using data from 769 adults with amnestic MCI who had participated in the ADC-008 trial of the ADCS [19]. As amnestic MCI is the most frequent phenotype of people with MCI due to AD [5], the findings of this validation study may be considered informative for those early AD patients with an amnestic MCI presentation.

While a single timepoint is frequently used to evaluate psychometric properties, we observed a somewhat narrow range of impairments in the population recruited into the ADC-008 study and valued including the more heterogenous and advanced MCI disease presentation observed in the cohort evaluated at the 36-month time point. Further, as evaluation of an instrument’s measurement properties is not related to the biologic reasons underlying disease process, we determined it appropriate to use both placebo- and experimental-treated patients at the 36-month timepoint. With the totality of these data, we found good evidence of psychometric validity for the ACDS-ADL-MCI in this cohort of amnestic MCI patients.

Item-level analysis indicated that ceiling effects on the ADCS-ADL-MCI total score were not apparent, while examining individual items indicated some isolated ceiling effects. Given the relatively mild condition of this cohort of patients, the findings are likely due to the relatively low impairment level of the cohort rather than the instrument's limitations. This is supported by the observation that fewer item-level ceiling effects were observed at month 36 compared to baseline. Interestingly, outcomes that may be attributed to sex differences were observed on some items, particularly the activities that may have sex-specific roles for some participants, as may be associated with the generation of patients represented in this study (e.g., cleaning and laundry). This finding suggests these questions were not assessing abilities but rather usual activities.

The ADCS-ADL-MCI demonstrated moderate evidence of internal consistency. While item-total correlations were overall acceptable at month 36, at baseline there were generally poor correlations for most items. These findings were anticipated as variability in responses are known to increase with increasing disease severity and items with higher response variability are expected to show stronger correlations with the total score [24, 25]. Of note, items with particularly low item-total correlations were the same items that also had lowest variability (e.g., items 2, 3, 17, and 18 at baseline).

Further, items with strong sex effects (e.g., items 4 and 7) also had low item-total correlations (< 0.30), indicating that they behave systematically different from the other items. Finally, the Cronbach’s alpha measure of internal consistency was acceptable, with a Cronbach's alpha of 0.64 at baseline, and 0.87 at month 36 [25]. The lack of variability in item responses at baseline likely similarly explains this difference in Cronbach’s alpha as well.

Reproducibility, defined by test–retest ICC, of the ADCS-ADL-MCI was moderate to good, especially as the timeframe between screening and baseline assessments (up to 6 months) was longer than typically desired (e.g., two weeks). In general, the ICC was higher when using the MCI-CGIC, GDS and CDR-SB vs. MMSE to define stability. One reason may be that the MMSE has higher variability in the MCI population than the other measure. Nonetheless, given the widespread use of the MMSE in this population, it was deemed important to explore 'stability' according to the MMSE as well.

The convergent validity of the ADCS-ADL-MCI with other relevant COA measures defined a priori showed moderate to high correlations. The ADCS-ADL-MCI was moderately correlated with the CDR-SB total score in the baseline measurement cohort. At month 36, strong correlations were observed between the ADCS-ADL-MCI total score and all the COA instruments evaluated, except for the BDI, Qol-AD-Informant total score, and NYU delayed paragraph recall test (moderate correlations observed). While a few of these results were contrary to expectations (i.e., we had expected weaker correlations with the NYU paragraph recall test and Symbol Digit Modalities Test), they indicate that functional, behavioral, and cognitive symptoms are all affected by (mild) cognitive impairment, particularly in a cohort with increased variability in disease presentation. Further, The ADCS-ADL-MCI likely had the strongest correlations with the CDR-SB as both measures assess functional aspects, whereas the MMSE and ADAS-Cog address predominantly cognitive aspects.

Further strong support was found for the known-groups validity of the ADCS-ADL-MCI, which is in line with previous publications [8, 16, 21]. Specifically, the ADCS-ADL-MCI was able to differentiate between disease severity groups as defined by the GDS, MMSE, BDI, and CDR-SB. Significant differences were observed among groups defined by *APOE* ε4 allele carrier status at month 36 but not baseline as expected, which confirms that the presence or absence of a biomarker when other clinical parameters are matched does not seem to differentiate ADL impairments in this early stage of the disease.

Finally, there was good support for the responsiveness of the ADCS-ADL-MCI, i.e., the instrument was adequately responsive in patients who indicated change in other instruments; and it was adequately stable in patients who did not show change in other instruments (CDR-SB and global CDR). Curiously, the 'improved' group according to CDR functional domain showed a marginal decrease in ADCS-ADL-MCI scores. Further, for the GDS, the two categories' improved' and 'no change' showed decreases in ADCS-ADL-MCI scores, with the former group showing even larger reductions than the latter group. Given that no confirmation of underlying AD or other neurodegenerative pathology was confirmed in this trial, it is plausible that some participants who meet criteria for MCI could subsequently “revert” to normal cognition. However, MCI patients reverting back to normal still remain at higher risk of progressing to dementia [1]. Further, it is possible that these findings reflect natural daily variations, differences in timepoints when the CDR-SB and ADL were measured, or a potential misclassification of patients as having improved but who were truly stable. Overall, there is evidence for the ability of the ADCS-ADL-MCI to detect a change when a change in the concept of interest has occurred.

Strengths of the study include a relatively large sample size that enabled us to carry out all psychometric tests planned and trust that results are robust, especially given that the sample was based on a diverse population in terms of age, education, and years since disease onset. Besides that, the ADC-008 trial dataset included an extensive range of COA instruments that enabled us to use these as reference instruments to assess various psychometric properties. Further, given that the study used data from an interventional clinical trial, tests of sensitivity to change over time were possible as the objective change had occurred in many trial participants over the 36-month duration of the trial.

The present study also has some limitations. For example, our target instrument was assessed at baseline, while some of the reference COA instruments had only been assessed at the screening assessment. Hence, while it is not expected that large changes occurred between these two time points, for the purpose of our project, it would have been preferable to have all COAs assessed at the same time point. However, we responded to this potential limitation by using 36-month data for our psychometric tests as well. The 36-month timepoint has limitations in that it only includes the aMCI patients who have not progressed to dementia, e.g., a more slowly progressing population. Thus, while the sensitivity to change reported herein may represent a conservative estimate, this reflects somewhat of a strength in that the psychometric property is shown in a more conservative sample. Given that *APOE* ε4 allele carriers are more likely to have underlying AD pathology and progress to dementia faster, it was of interest to examine whether there were more or less carriers at this timepoint. The proportion of APOE4 carriers was similar at baseline and month 36.

Further, the two-step response process to most ADCS-ADL-MCI items poses some challenges for psychometric evaluation. For example, scale-level tests such as factor analysis on gated items are more challenging than those on questions with thoroughly graded responses. Therefore, we could not confirm nor deny the unidimensionality assumption of the ADCS-ADL-MCI. However, our results suggest that the ADCS-ADL-MCI performs well across multiple psychometric tests despite this limitation. The diversitiy of the enrolled clinical cohort was poor, with 92% of the cohort “white, not of Hispanic Origin”. While the inclusion criteria for the study was extensive to confirm amnestic MCI, subjects were not required to have evidence of amyloid or tau pathology, thus the application of these findings to MCI patients on the AD continuum can be considered an extrapolation on the basis that a clinical diagnosis of amnestic MCI is the same regardless of the underlying disease pathology. It is nonetheless important to note that patients with MCI may not necessarily have underlying AD. Finally, a caveat to the use of informant-based methods is reliance on the caregiver’s knowledge about patient’s daily activities. As such, the accuracy of these reports is somewhat limited to the amount of care provided by the informant.

Overall, findings from this study suggest that the ADCS-ADL-MCI is reliable, valid and responsive to change in an amnestic MCI population. However, further investigation is needed in this sample, and a comparison of performance-based measures, self-report and informant-report measures may help define functional impairment in MCI (in terms of quanitifable cut-off scores) more precisely.

## Conclusions

In conclusion, this study provides supportive evidence that the ADCS-ADL-MCI is reliable, with acceptable internal consistency (item-total correlations and Cronbach’s alphas) and moderate-to-good reproducibility. Convergent validity was demonstrated, with moderate-to-high correlations observed between the ADCS-ADL-MCI with other relevant COA measures. Further, there was strong support for known-groups validity, as evidenced by the ADCS-ADL-MCI’s ability to distinguish between various disease severity groups. Finally, the ADCS-ADL-MCI was shown to be responsive to change when patients reported change on other COA measures. This instrumentis fit-for-purpose as a key endpoint in pivotal trials and for use by clinicians in clinical practice, capable of capturing amnestic MCI patients' functional abilities in ADLs from a caregiver's perspective.

## Supplementary Information

**Additional file 1: Supplemental Table 1.** Distribution of ADCS-ADL-MCI individual item scores at baseline by sex. **Supplemental Table 2.** Distribution of ADCS-ADL-MCI individual item scores at month 36 by sex.

## Data Availability

All results generated or analysed during this study are included in this published article and its supplementary information files. The source data used to develop the results are available from ADCS with permission of ADCS.

## Abbreviations

AD: Alzheimer’s Disease
ADAS-Cog 13: Alzheimer’s Disease Assessment Scale Cognitive Subscale
ADCS: Alzheimer’s Disease Cooperative Study
ADCS-ADL-MCI: Alzheimer's Disease Cooperative Study—Activities of Daily Living scale for use in Mild Cognitive Impairment
ADLs: Activities of Daily Living
ANOVA: Analysis of Variance
APOE: Apolipoprotein epsilon 4
BADLs: Basic Activities of Daily Living
BDI: Beck Depression Inventory
CDR-SB: Clinical Dementia Rating – Sum of Boxes
COA: Clinical Outcomes Assessment
ES: Effect Size
GDS: Global Deterioration Scale
H_0_: Null Hypothesis
H_1_: Alternative Hypothesis
iADLs: Instrumental Activities of Daily Living
ICC: Intraclass Correlation Coefficient
IQR: Interquartile Range
MCI: Mild Cognitive Impairment
MCI-CGIC: Mild Cognitive Impairment – Clinician Global Impression of Change
MMSE: Mini-Mental State Examination
QoL: Quality of Life
QoL-AD: Quality of Life – Alzheimer’s Disease
SAP: Statistical Analysis Plan
SD: Standard Deviation
SE: Standard Error
