# Supplementary material for: Psychometric properties of the Alzheimer’s Disease Cooperative Study – Activities of Daily Living for Mild Cognitive Impairment (ADCS-MCI-ADL) scale: a post hoc analysis of the ADCS ADC-008 trial
Source: BMC Geriatr. 2023 Mar 6;23:124. doi: 10.1186/s12877-022-03527-0 (PMC9990271; doi:10.1186/s12877-022-03527-0)
Supplement: Supplementary file 1 — Additional file 1: Supplemental Table 1. Distribution of ADCS-ADL-MCI individual item scores at baseline by sex. Supplemental Table 2. Distribution of ADCS-ADL-MCI individual item scores at month 36 by sex. [file 12877_2022_3527_MOESM1_ESM.docx]

| **Supplemental Table 1.** Distribution of ADCS-ADL-MCI individual item scores at baseline by sex | | | | | | |
| --- | --- | --- | --- | --- | --- | --- |
|  | Overall | | Male | | Female | |
| ADCS-ADL-MCI Item | N=769 | % | N=417 | % | N=352 | % |
| Item 1: In the past 4 weeks, did (S) usually manage to find his/her personal belongings at home? |  |  |  |  |  |  |
| 1=Yes | 748 | 97.27 | 404 | 96.88 | 344 | 97.73 |
| 2=No | 18 | 2.34 | 10 | 2.4 | 8 | 2.27 |
| 3=I don’t know | 2 | 0.26 | 2 | 0.48 | 0 | 0 |
| Missing | 1 | 0.13 | 1 | 0.24 | 0 | 0 |
| If yes, which best describes how he/she usually performed: |  |  |  |  |  |  |
| 1=With physical help | 14 | 1.82 | 11 | 2.64 | 3 | 0.85 |
| 2=With supervision | 94 | 12.22 | 53 | 12.71 | 41 | 11.65 |
| 3=Without supervision or help | 640 | 83.22 | 340 | 81.53 | 300 | 85.23 |
| Missing | 21 | 2.73 | 13 | 3.12 | 8 | 2.27 |
| Item 2: In the past 4 weeks, did (S) select his/her first set of clothes for the day? |  |  |  |  |  |  |
| 1=Yes | 762 | 99.09 | 413 | 99.04 | 349 | 99.15 |
| 2=No | 4 | 0.52 | 2 | 0.48 | 2 | 0.57 |
| 3=I don’t know | 2 | 0.26 | 1 | 0.24 | 1 | 0.28 |
| Missing | 1 | 0.13 | 1 | 0.24 | 0 | 0 |
| If yes, which best describes his/her usual performance: |  |  |  |  |  |  |
| 1=With physical help | 3 | 0.39 | 3 | 0.72 | 0 | 0 |
| 2=With supervision | 25 | 3.25 | 23 | 5.52 | 2 | 0.57 |
| 3=Without supervision or help | 734 | 95.45 | 387 | 92.81 | 347 | 98.58 |
| Missing | 7 | 0.91 | 4 | 0.96 | 3 | 0.85 |
| Item 3: Regarding physically getting dressed, which best describes his/her usual performance in the past 4 weeks? |  |  |  |  |  |  |
| 0=Someone else dressed him/her | 0 | 0 | 0 | 0 | 0 | 0 |
| 1=Needed some help even if clothes needed no fastening or buttoning | 0 | 0 | 0 | 0 | 0 | 0 |
| 2=Need physical help only for buttons, clasps or shoelaces | 2 | 0.26 | 0 | 0 | 2 | 0.57 |
| 3=Dressed completely with supervision, but without help | 4 | 0.52 | 2 | 0.48 | 2 | 0.57 |
| 4=Dressed completely without supervision or physical help | 761 | 98.96 | 414 | 99.28 | 347 | 98.58 |
| Missing | 2 | 0.26 | 1 | 0.24 | 1 | 0.28 |
| Item 4: In the past 4 weeks, did (S) clean a living, sitting or family room? |  |  |  |  |  |  |
| 1=Yes | 586 | 76.2 | 278 | 66.67 | 308 | 87.5 |
| 2=No | 176 | 22.89 | 135 | 32.37 | 41 | 11.65 |
| 3=I don’t know | 6 | 0.78 | 3 | 0.72 | 3 | 0.85 |
| Missing | 1 | 0.13 | 1 | 0.24 | 0 | 0 |
| If yes, which best describes how he/she usually performed? |  |  |  |  |  |  |
| 0=With physical help | 7 | 0.91 | 0 | 0 | 7 | 1.99 |
| 1=With supervision | 11 | 1.43 | 7 | 1.68 | 4 | 1.14 |
| 2=Without supervision or help | 568 | 73.86 | 271 | 64.99 | 297 | 84.38 |
| Missing | 183 | 23.8 | 139 | 33.33 | 44 | 12.5 |
| Item 5: In the past 4 weeks, did (S) balance his/her checkbook or a credit card statement? |  |  |  |  |  |  |
| 1=Yes | 488 | 63.46 | 247 | 59.23 | 241 | 68.47 |
| 2=No | 263 | 34.2 | 159 | 38.13 | 104 | 29.55 |
| 3=I don’t know | 17 | 2.21 | 10 | 2.4 | 7 | 1.99 |
| Missing | 1 | 0.13 | 1 | 0.24 | 0 | 0 |
| If yes, which best describes how he/she usually performed? |  |  |  |  |  |  |
| 0=With physical help | 7 | 0.91 | 3 | 0.72 | 4 | 1.14 |
| 1=With supervision | 56 | 7.28 | 21 | 5.04 | 35 | 9.94 |
| 2=Without supervision or help | 425 | 55.27 | 223 | 53.48 | 202 | 57.39 |
| Missing | 281 | 36.54 | 170 | 40.77 | 111 | 31.53 |
| Item 6: In the past 4 weeks, did (S) ever write things down? |  |  |  |  |  |  |
| 1=Yes | 745 | 96.88 | 401 | 96.16 | 344 | 97.73 |
| 2=No | 20 | 2.6 | 13 | 3.12 | 7 | 1.99 |
| 3=I don’t know | 3 | 0.39 | 2 | 0.48 | 1 | 0.28 |
| Missing | 1 | 0.13 | 1 | 0.24 | 0 | 0 |
| If yes, which best describes the most complicated things that he/she wrote? |  |  |  |  |  |  |
| 0=His/her signature or name | 23 | 2.99 | 19 | 4.56 | 4 | 1.14 |
| 1=Short notes or messages that other people understood | 377 | 49.02 | 208 | 49.88 | 169 | 48.01 |
| 2=Letters or long notes that other people understood | 345 | 44.86 | 174 | 41.73 | 171 | 48.58 |
| Missing | 24 | 3.12 | 16 | 3.84 | 8 | 2.27 |
| Item 7: In the past 4 weeks, did (S) clean a load of laundry? |  |  |  |  |  |  |
| 1=Yes | 524 | 68.14 | 186 | 44.6 | 338 | 96.02 |
| 2=No | 241 | 31.34 | 227 | 54.44 | 14 | 3.98 |
| 3=I don’t know | 3 | 0.39 | 3 | 0.72 | 0 | 0 |
| Missing | 1 | 0.13 | 1 | 0.24 | 0 | 0 |
| If yes, which best describes how he/she usually performed: |  |  |  |  |  |  |
| 0=With physical help | 2 | 0.26 | 0 | 0 | 2 | 0.57 |
| 1=With supervision | 9 | 1.17 | 5 | 1.2 | 4 | 1.14 |
| 2=Without supervision or help | 513 | 66.71 | 181 | 43.41 | 332 | 94.32 |
| Missing | 245 | 31.86 | 231 | 55.4 | 14 | 3.98 |
| Item 8: In the past 4 weeks, did (S) keep appointments or meetings with other people, such as relatives, a doctor, the hairdresser, etc.? |  |  |  |  |  |  |
| 1=Yes | 761 | 98.96 | 411 | 98.56 | 350 | 99.43 |
| 2=No | 6 | 0.78 | 4 | 0.96 | 2 | 0.57 |
| 3=I don’t know | 1 | 0.13 | 1 | 0.24 | 0 | 0 |
| Missing | 1 | 0.13 | 1 | 0.24 | 0 | 0 |
| If yes, which best describes his/her awareness of the meeting ahead of time: |  |  |  |  |  |  |
| 0=Usually did not remember, in spite of verbal reminders on the day | 6 | 0.78 | 5 | 1.2 | 1 | 0.28 |
| 1=Usually remembered the appointment after verbal reminders on the day | 108 | 14.04 | 76 | 18.23 | 32 | 9.09 |
| 2=Usually referred to notes, a diary, or calendar | 539 | 70.09 | 270 | 64.75 | 269 | 76.42 |
| 3=Usually remembered without written or verbal reminders | 108 | 14.04 | 60 | 14.39 | 48 | 13.64 |
| Missing | 8 | 1.04 | 6 | 1.44 | 2 | 0.57 |
| Item 9: In the past 4 weeks, did (S) use a telephone? |  |  |  |  |  |  |
| 1=Yes | 767 | 99.74 | 415 | 99.52 | 352 | 100 |
| 2=No | 0 | 0 | 0 | 0 | 0 | 0 |
| 3=I don’t know | 1 | 0.13 | 1 | 0.24 | 0 | 0 |
| Missing | 1 | 0.13 | 1 | 0.24 | 0 | 0 |
| If yes, which best describes his/her highest level of performance: |  |  |  |  |  |  |
| 0=Did not answer the phone, but spoke when put on the line | 1 | 0.13 | 1 | 0.24 | 0 | 0 |
| 1=Answered the phone and spoke to callers; did not make calls | 8 | 1.04 | 6 | 1.44 | 2 | 0.57 |
| 2=Made calls only to well-known numbers, by using a directory or list | 76 | 9.88 | 40 | 9.59 | 36 | 10.23 |
| 3=Made calls only to well-known numbers, without referring to a directory or list | 81 | 10.53 | 39 | 9.35 | 42 | 11.93 |
| 4=Made any call necessary e.g., after looking up numbers in white or yellow pages, or by dialing directory assistance | 601 | 78.15 | 329 | 78.9 | 272 | 77.27 |
| Missing | 2 | 0.26 | 2 | 0.48 | 0 | 0 |
| Item 10: In the past 4 weeks, did (S) make him/herself a meal or snack at home? |  |  |  |  |  |  |
| 1=Yes | 750 | 97.53 | 401 | 96.16 | 349 | 99.15 |
| 2=No | 18 | 2.34 | 15 | 3.6 | 3 | 0.85 |
| 3=I don’t know | 0 | 0 | 0 | 0 | 0 | 0 |
| Missing | 1 | 0.13 | 1 | 0.24 | 0 | 0 |
| If yes, which best describes his/her highest level of food preparation: |  |  |  |  |  |  |
| 0=Obtained food on his/her own, without mixing or cooking it | 13 | 1.69 | 12 | 2.88 | 1 | 0.28 |
| 1=Mixed or combined food items for a meal or snack, without cooking or microwaving (e.g. made a sandwich) | 48 | 6.24 | 43 | 10.31 | 5 | 1.42 |
| 2=Cooked or microwaved food, with extensive help | 10 | 1.3 | 5 | 1.2 | 5 | 1.42 |
| 3=Cooked or microwaved food, with little or no help | 679 | 88.3 | 341 | 81.77 | 338 | 96.02 |
| Missing | 19 | 2.47 | 16 | 3.84 | 3 | 0.85 |
| Item 11: In the past 4 weeks, did (S) get around (or travel) outside of his/her home? |  |  |  |  |  |  |
| 1=Yes | 764 | 99.35 | 416 | 99.76 | 348 | 98.86 |
| 2=No | 4 | 0.52 | 0 | 0 | 4 | 1.14 |
| 3=I don’t know | 0 | 0 | 0 | 0 | 0 | 0 |
| Missing | 1 | 0.13 | 1 | 0.24 | 0 | 0 |
| If yes, which best describes his/her optimal performance: |  |  |  |  |  |  |
| 0=Travelled only with physical help, regardless of the trip | 2 | 0.26 | 0 | 0 | 2 | 0.57 |
| 1=Travelled only with a chaperone for supervision regardless of the trip | 30 | 3.9 | 7 | 1.68 | 23 | 6.53 |
| 2=Travelled alone, but remained within 1 mile of home | 28 | 3.64 | 10 | 2.4 | 18 | 5.11 |
| 3=Travelled alone, went at least 1 mile away from home | 704 | 91.55 | 399 | 95.68 | 305 | 86.65 |
| Missing | 5 | 0.65 | 1 | 0.24 | 4 | 1.14 |
| Item 12: In the past 4 weeks, did (S) talk about current events? |  |  |  |  |  |  |
| 1=Yes | 753 | 97.92 | 410 | 98.32 | 343 | 97.44 |
| 2=No | 15 | 1.95 | 6 | 1.44 | 9 | 2.56 |
| 3=I don’t know | 0 | 0 | 0 | 0 | 0 | 0 |
| Missing | 1 | 0.13 | 1 | 0.24 | 0 | 0 |
| Item 12a: Did (S) talk about regional, national or international events (including sports)? |  |  |  |  |  |  |
| 0=No | 38 | 4.94 | 17 | 4.08 | 21 | 5.97 |
| 1=Yes | 715 | 92.98 | 393 | 94.24 | 322 | 91.48 |
| Missing | 16 | 2.08 | 7 | 1.68 | 9 | 2.56 |
| Item 12b: Did (S) talk about events outside home involving family, friends or neighbors? |  |  |  |  |  |  |
| 0=No | 24 | 3.12 | 18 | 4.32 | 6 | 1.7 |
| 1=Yes | 729 | 94.8 | 392 | 94 | 337 | 95.74 |
| Missing | 16 | 2.08 | 7 | 1.68 | 9 | 2.56 |
| Item 12c: Did (S) talk about events that occurred at home that he/she took part in or watched? |  |  |  |  |  |  |
| 0=No | 40 | 5.2 | 27 | 6.47 | 13 | 3.69 |
| 1=Yes | 713 | 92.72 | 383 | 91.85 | 330 | 93.75 |
| Missing | 16 | 2.08 | 7 | 1.68 | 9 | 2.56 |
| Item 12d: Did (S) converse without repeating him/herself, or asking the same questions repeatedly? |  |  |  |  |  |  |
| 0=No | 266 | 34.59 | 131 | 31.41 | 135 | 38.35 |
| 1=Yes | 487 | 63.33 | 279 | 66.91 | 208 | 59.09 |
| Missing | 16 | 2.08 | 7 | 1.68 | 9 | 2.56 |
| Item 13: In the past 4 weeks, did (S) read a magazine, newspaper or book for more than 5 minutes at a time? |  |  |  |  |  |  |
| 1=Yes | 746 | 97.01 | 405 | 97.12 | 341 | 96.88 |
| 2=No | 22 | 2.86 | 11 | 2.64 | 11 | 3.13 |
| 3=I don’t know | 0 | 0 | 0 | 0 | 0 | 0 |
| Missing | 1 | 0.13 | 1 | 0.24 | 0 | 0 |
| Item 13a: Did (S) usually select or ask for something to read? |  |  |  |  |  |  |
| 0=No | 20 | 2.6 | 12 | 2.88 | 8 | 2.27 |
| 1=Yes | 726 | 94.41 | 393 | 94.24 | 333 | 94.6 |
| Missing | 23 | 2.99 | 12 | 2.88 | 11 | 3.13 |
| Item 13b: Did (S) usually talk about what he/she read while or shortly after reading (less than 1 hour)? |  |  |  |  |  |  |
| 0=No | 157 | 20.42 | 87 | 20.86 | 70 | 19.89 |
| 1=Yes | 589 | 76.59 | 318 | 76.26 | 271 | 76.99 |
| Missing | 23 | 2.99 | 12 | 2.88 | 11 | 3.13 |
| Item 13c: Did (S) usually talk about what he/she read while 1-24 hours after reading? |  |  |  |  |  |  |
| 0=No | 269 | 34.98 | 152 | 36.45 | 117 | 33.24 |
| 1=Yes | 477 | 62.03 | 253 | 60.67 | 224 | 63.64 |
| Missing | 23 | 2.99 | 12 | 2.88 | 11 | 3.13 |
| Item 14: In the past 4 weeks, did (S) watch television? |  |  |  |  |  |  |
| 1=Yes | 755 | 98.18 | 409 | 98.08 | 346 | 98.3 |
| 2=No | 12 | 1.56 | 7 | 1.68 | 5 | 1.42 |
| 3=I don't know | 1 | 0.13 | 0 | 0 | 1 | 0.28 |
| Missing | 1 | 0.13 | 1 | 0.24 | 0 | 0 |
| Item 14a: Did (S) usually select or ask for different programs or his/her favorite show? |  |  |  |  |  |  |
| 0=No | 39 | 5.07 | 17 | 4.08 | 22 | 6.25 |
| 1=Yes | 716 | 93.11 | 392 | 94 | 324 | 92.05 |
| Missing | 14 | 1.82 | 8 | 1.92 | 6 | 1.7 |
| Item 14b: Did (S) usually talk about the content of a program while watching it? |  |  |  |  |  |  |
| 0=No | 170 | 22.11 | 92 | 22.06 | 78 | 22.16 |
| 1=Yes | 585 | 76.07 | 317 | 76.02 | 268 | 76.14 |
| Missing | 14 | 1.82 | 8 | 1.92 | 6 | 1.7 |
| Item 14c: Did (S) talk about the content of a program within a day (24 hours) after watching it? |  |  |  |  |  |  |
| 0=No | 283 | 36.8 | 158 | 37.89 | 125 | 35.51 |
| 1=Yes | 472 | 61.38 | 251 | 60.19 | 221 | 62.78 |
| Missing | 14 | 1.82 | 8 | 1.92 | 6 | 1.7 |
| Item 15: In the past 4 weeks, did (S) ever go shopping at a store? |  |  |  |  |  |  |
| 1=Yes | 754 | 98.05 | 408 | 97.84 | 346 | 98.3 |
| 2=No | 14 | 1.82 | 8 | 1.92 | 6 | 1.7 |
| 3=I don’t know | 0 | 0 | 0 | 0 | 0 | 0 |
| Missing | 1 | 0.13 | 1 | 0.24 | 0 | 0 |
| Item 15a: Did (S) usually select correct items without supervision or help? |  |  |  |  |  |  |
| 0=No | 33 | 4.29 | 22 | 5.28 | 11 | 3.13 |
| 1=Yes | 721 | 93.76 | 386 | 92.57 | 335 | 95.17 |
| Missing | 15 | 1.95 | 9 | 2.16 | 6 | 1.7 |
| Item 15b: Did (S) usually pay for items on his/her own? |  |  |  |  |  |  |
| 0=No | 30 | 3.9 | 15 | 3.6 | 15 | 4.26 |
| 1=Yes | 724 | 94.15 | 393 | 94.24 | 331 | 94.03 |
| Missing | 15 | 1.95 | 9 | 2.16 | 6 | 1.7 |
| Item 16: In the past 4 weeks, was (S) ever left on his/her own? |  |  |  |  |  |  |
| 1=Yes | 762 | 99.09 | 414 | 99.28 | 348 | 98.86 |
| 2=No | 6 | 0.78 | 2 | 0.48 | 4 | 1.14 |
| 3=I don’t know | 0 | 0 | 0 | 0 | 0 | 0 |
| Missing | 1 | 0.13 | 1 | 0.24 | 0 | 0 |
| Item 16a: Was (S) left away from home, for 15 minutes or longer, during the day? |  |  |  |  |  |  |
| 0=No | 32 | 4.16 | 16 | 3.84 | 16 | 4.55 |
| 1=Yes | 730 | 94.93 | 398 | 95.44 | 332 | 94.32 |
| Missing | 7 | 0.91 | 3 | 0.72 | 4 | 1.14 |
| Item 16b: Was (S) left at home, for an hour or longer, during the day? |  |  |  |  |  |  |
| 0=No | 10 | 1.3 | 3 | 0.72 | 7 | 1.99 |
| 1=Yes | 752 | 97.79 | 411 | 98.56 | 341 | 96.88 |
| Missing | 7 | 0.91 | 3 | 0.72 | 4 | 1.14 |
| Item 16c: Was (S) left at home, for less than 1 hour, during the day? |  |  |  |  |  |  |
| 0=No | 42 | 5.46 | 19 | 4.56 | 23 | 6.53 |
| 1=Yes | 720 | 93.63 | 395 | 94.72 | 325 | 92.33 |
| Missing | 7 | 0.91 | 3 | 0.72 | 4 | 1.14 |
| Item 17: In the past 4 weeks, did (S) use a household appliance to do chores? |  |  |  |  |  |  |
| 1=Yes | 760 | 98.83 | 409 | 98.08 | 351 | 99.72 |
| 2=No | 6 | 0.78 | 5 | 1.2 | 1 | 0.28 |
| 3=I don’t know | 2 | 0.26 | 2 | 0.48 | 0 | 0 |
| Missing | 1 | 0.13 | 1 | 0.24 | 0 | 0 |
| If yes, check all that apply: |  |  |  |  |  |  |
| Washer |  |  |  |  |  |  |
| Box Checked | 512 | 66.58 | 182 | 43.65 | 330 | 93.75 |
| Box Not Checked | 248 | 32.25 | 227 | 54.44 | 21 | 5.97 |
| Missing | 9 | 1.17 | 8 | 1.92 | 1 | 0.28 |
| Dishwasher |  |  |  |  |  |  |
| Box Checked | 434 | 56.44 | 197 | 47.24 | 237 | 67.33 |
| Box Not Checked | 326 | 42.39 | 212 | 50.84 | 114 | 32.39 |
| Missing | 9 | 1.17 | 8 | 1.92 | 1 | 0.28 |
| Range |  |  |  |  |  |  |
| Box Checked | 607 | 78.93 | 282 | 67.63 | 325 | 92.33 |
| Box Not Checked | 153 | 19.9 | 127 | 30.46 | 26 | 7.39 |
| Missing | 9 | 1.17 | 8 | 1.92 | 1 | 0.28 |
| Dryer |  |  |  |  |  |  |
| Box Checked | 509 | 66.19 | 192 | 46.04 | 317 | 90.06 |
| Box Not Checked | 251 | 32.64 | 217 | 52.04 | 34 | 9.66 |
| Missing | 9 | 1.17 | 8 | 1.92 | 1 | 0.28 |
| Power tool(s) |  |  |  |  |  |  |
| Box Checked | 220 | 28.61 | 205 | 49.16 | 15 | 4.26 |
| Box Not Checked | 540 | 70.22 | 204 | 48.92 | 336 | 95.45 |
| Missing | 9 | 1.17 | 8 | 1.92 | 1 | 0.28 |
| Microwave |  |  |  |  |  |  |
| Box Checked | 672 | 87.39 | 357 | 85.61 | 315 | 89.49 |
| Box Not Checked | 88 | 11.44 | 52 | 12.47 | 36 | 10.23 |
| Missing | 9 | 1.17 | 8 | 1.92 | 1 | 0.28 |
| Vacuum |  |  |  |  |  |  |
| Box Checked | 524 | 68.14 | 269 | 64.51 | 255 | 72.44 |
| Box Not Checked | 236 | 30.69 | 140 | 33.57 | 96 | 27.27 |
| Missing | 9 | 1.17 | 8 | 1.92 | 1 | 0.28 |
| Toaster oven |  |  |  |  |  |  |
| Box Checked | 443 | 57.61 | 216 | 51.8 | 227 | 64.49 |
| Box Not Checked | 317 | 41.22 | 193 | 46.28 | 124 | 35.23 |
| Missing | 9 | 1.17 | 8 | 1.92 | 1 | 0.28 |
| Food processor |  |  |  |  |  |  |
| Box Checked | 121 | 15.73 | 20 | 4.8 | 101 | 28.69 |
| Box Not Checked | 639 | 83.09 | 389 | 93.29 | 250 | 71.02 |
| Missing | 9 | 1.17 | 8 | 1.92 | 1 | 0.28 |
| Other |  |  |  |  |  |  |
| Box Checked | 204 | 26.53 | 107 | 25.66 | 97 | 27.56 |
| Box Not Checked | 556 | 72.3 | 302 | 72.42 | 254 | 72.16 |
| Missing | 9 | 1.17 | 8 | 1.92 | 1 | 0.28 |
| Item 17a: For the 1 or 2 most commonly used appliances, which best describes how (S) usually used them: |  |  |  |  |  |  |
| 1=With physical help | 0 | 0 | 0 | 0 | 0 | 0 |
| 2=With supervision (e.g., instructions), but no physical help | 6 | 0.78 | 5 | 1.2 | 1 | 0.28 |
| 3=Without help, but operated only on/off controls | 13 | 1.69 | 9 | 2.16 | 4 | 1.14 |
| 4=Without help, and operated all necessary controls | 741 | 96.36 | 395 | 94.72 | 346 | 98.3 |
| Missing | 9 | 1.17 | 8 | 1.92 | 1 | 0.28 |
| Item 18: In the past 4 weeks, did (S) perform a pastime, hobby or game? |  |  |  |  |  |  |
| 1=Yes | 739 | 96.1 | 400 | 95.92 | 339 | 96.31 |
| 2=No | 27 | 3.51 | 15 | 3.6 | 12 | 3.41 |
| 3=I don’t know | 2 | 0.26 | 1 | 0.24 | 1 | 0.28 |
| Missing | 1 | 0.13 | 1 | 0.24 | 0 | 0 |
| If yes, check all that apply: |  |  |  |  |  |  |
| Card or board games |  |  |  |  |  |  |
| Box Checked | 262 | 34.07 | 136 | 32.61 | 126 | 35.8 |
| Box Not Checked | 477 | 62.03 | 264 | 63.31 | 213 | 60.51 |
| Missing | 30 | 3.9 | 17 | 4.08 | 13 | 3.69 |
| Bingo |  |  |  |  |  |  |
| Box Checked | 32 | 4.16 | 10 | 2.4 | 22 | 6.25 |
| Box Not Checked | 707 | 91.94 | 390 | 93.53 | 317 | 90.06 |
| Missing | 30 | 3.9 | 17 | 4.08 | 13 | 3.69 |
| Musical instrument |  |  |  |  |  |  |
| Box Checked | 44 | 5.72 | 24 | 5.76 | 20 | 5.68 |
| Box Not Checked | 695 | 90.38 | 376 | 90.17 | 319 | 90.63 |
| Missing | 30 | 3.9 | 17 | 4.08 | 13 | 3.69 |
| Reading |  |  |  |  |  |  |
| Box Checked | 599 | 77.89 | 319 | 76.5 | 280 | 79.55 |
| Box Not Checked | 140 | 18.21 | 81 | 19.42 | 59 | 16.76 |
| Missing | 30 | 3.9 | 17 | 4.08 | 13 | 3.69 |
| Tennis |  |  |  |  |  |  |
| Box Checked | 32 | 4.16 | 22 | 5.28 | 10 | 2.84 |
| Box Not Checked | 707 | 91.94 | 378 | 90.65 | 329 | 93.47 |
| Missing | 30 | 3.9 | 17 | 4.08 | 13 | 3.69 |
| Crosswords |  |  |  |  |  |  |
| Box Checked | 177 | 23.02 | 74 | 17.75 | 103 | 29.26 |
| Box Not Checked | 562 | 73.08 | 326 | 78.18 | 236 | 67.05 |
| Missing | 30 | 3.9 | 17 | 4.08 | 13 | 3.69 |
| Knitting |  |  |  |  |  |  |
| Box Checked | 36 | 4.68 | 1 | 0.24 | 35 | 9.94 |
| Box Not Checked | 703 | 91.42 | 399 | 95.68 | 304 | 86.36 |
| Missing | 30 | 3.9 | 17 | 4.08 | 13 | 3.69 |
| Gardening |  |  |  |  |  |  |
| Box Checked | 294 | 38.23 | 140 | 33.57 | 154 | 43.75 |
| Box Not Checked | 445 | 57.87 | 260 | 62.35 | 185 | 52.56 |
| Missing | 30 | 3.9 | 17 | 4.08 | 13 | 3.69 |
| Workshop |  |  |  |  |  |  |
| Box Checked | 127 | 16.51 | 122 | 29.26 | 5 | 1.42 |
| Box Not Checked | 612 | 79.58 | 278 | 66.67 | 334 | 94.89 |
| Missing | 30 | 3.9 | 17 | 4.08 | 13 | 3.69 |
| Art |  |  |  |  |  |  |
| Box Checked | 28 | 3.64 | 10 | 2.4 | 18 | 5.11 |
| Box Not Checked | 711 | 92.46 | 390 | 93.53 | 321 | 91.19 |
| Missing | 30 | 3.9 | 17 | 4.08 | 13 | 3.69 |
| Sewing |  |  |  |  |  |  |
| Box Checked | 89 | 11.57 | 2 | 0.48 | 87 | 24.72 |
| Box Not Checked | 650 | 84.53 | 398 | 95.44 | 252 | 71.59 |
| Missing | 30 | 3.9 | 17 | 4.08 | 13 | 3.69 |
| Golf |  |  |  |  |  |  |
| Box Checked | 96 | 12.48 | 76 | 18.23 | 20 | 5.68 |
| Box Not Checked | 643 | 83.62 | 324 | 77.7 | 319 | 90.63 |
| Missing | 30 | 3.9 | 17 | 4.08 | 13 | 3.69 |
| Fishing |  |  |  |  |  |  |
| Box Checked | 16 | 2.08 | 15 | 3.6 | 1 | 0.28 |
| Box Not Checked | 723 | 94.02 | 385 | 92.33 | 338 | 96.02 |
| Missing | 30 | 3.9 | 17 | 4.08 | 13 | 3.69 |
| Other |  |  |  |  |  |  |
| Box Checked | 314 | 40.83 | 183 | 43.88 | 131 | 37.22 |
| Box Not Checked | 425 | 55.27 | 217 | 52.04 | 208 | 59.09 |
| Missing | 30 | 3.9 | 17 | 4.08 | 13 | 3.69 |
| Item 18a: Did (S) require supervision, or help, to perform any of these hobbies? |  |  |  |  |  |  |
| 1=Help | 2 | 0.26 | 1 | 0.24 | 1 | 0.28 |
| 2=Supervision | 6 | 0.78 | 4 | 0.96 | 2 | 0.57 |
| 3=No supervision required | 731 | 95.06 | 395 | 94.72 | 336 | 95.45 |
| Missing | 30 | 3.9 | 17 | 4.08 | 13 | 3.69 |

| **Supplemental Table 2.** Distribution of ADCS-ADL-MCI individual item scores at month 36 by sex | | | | | | |
| --- | --- | --- | --- | --- | --- | --- |
|  | Overall | | Male | | Female | |
| ADCS-ADL-MCI Item | N=484 | % | N=277 | % | N=207 | % |
| Item 1: In the past 4 weeks, did (S) usually manage to find his/her personal belongings at home? |  |  |  |  |  |  |
| 1=Yes | 460 | 95.04 | 265 | 95.67 | 195 | 94.2 |
| 2=No | 14 | 2.89 | 7 | 2.53 | 7 | 3.38 |
| 3=I don't know | 3 | 0.62 | 0 | 0 | 3 | 1.45 |
| Missing | 7 | 1.45 | 5 | 1.81 | 2 | 0.97 |
| If yes, which best describes how he/she usually performed: |  |  |  |  |  |  |
| 1=With physical help | 39 | 8.06 | 22 | 7.94 | 17 | 8.21 |
| 2=With supervision | 79 | 16.32 | 44 | 15.88 | 35 | 16.91 |
| 3=Without supervision or help | 342 | 70.66 | 199 | 71.84 | 143 | 69.08 |
| Missing | 24 | 4.96 | 12 | 4.33 | 12 | 5.8 |
| Item 2: In the past 4 weeks, did (S) select his/her first set of clothes for the day? |  |  |  |  |  |  |
| 1=Yes | 466 | 96.28 | 265 | 95.67 | 201 | 97.1 |
| 2=No | 10 | 2.07 | 7 | 2.53 | 3 | 1.45 |
| 3=I don't know | 1 | 0.21 | 0 | 0 | 1 | 0.48 |
| Missing | 7 | 1.45 | 5 | 1.81 | 2 | 0.97 |
| If yes, which best describes his/her usual performance: |  |  |  |  |  |  |
| 1=With physical help | 12 | 2.48 | 5 | 1.81 | 7 | 3.38 |
| 2=With supervision | 42 | 8.68 | 32 | 11.55 | 10 | 4.83 |
| 3=Without supervision or help | 412 | 85.12 | 228 | 82.31 | 184 | 88.89 |
| Missing | 18 | 3.72 | 12 | 4.33 | 6 | 2.9 |
| Item 3: Regarding physically getting dressed, which best describes his/her usual performance in the past 4 weeks? |  |  |  |  |  |  |
| 0=Someone else dressed him/her | 3 | 0.62 | 1 | 0.36 | 2 | 0.97 |
| 1=Needed some help even if clothes needed no fastening or buttoning | 10 | 2.07 | 3 | 1.08 | 7 | 3.38 |
| 2=Need physical help only for buttons, clasps or shoelaces | 9 | 1.86 | 4 | 1.44 | 5 | 2.42 |
| 3=Dressed completely with supervision, but without help | 13 | 2.69 | 9 | 3.25 | 4 | 1.93 |
| 4=Dressed completely without supervision or physical help | 441 | 91.12 | 254 | 91.7 | 187 | 90.34 |
| Missing | 8 | 1.65 | 6 | 2.17 | 2 | 0.97 |
| Item 4: In the past 4 weeks, did (S) clean a living, sitting or family room? |  |  |  |  |  |  |
| 1=Yes | 333 | 68.8 | 176 | 63.54 | 157 | 75.85 |
| 2=No | 141 | 29.13 | 96 | 34.66 | 45 | 21.74 |
| 3=I don't know | 3 | 0.62 | 0 | 0 | 3 | 1.45 |
| Missing | 7 | 1.45 | 5 | 1.81 | 2 | 0.97 |
| If yes, which best describes how he/she usually performed? |  |  |  |  |  |  |
| 0=With physical help | 6 | 1.24 | 2 | 0.72 | 4 | 1.93 |
| 1=With supervision | 33 | 6.82 | 20 | 7.22 | 13 | 6.28 |
| 2=Without supervision or help | 294 | 60.74 | 154 | 55.6 | 140 | 67.63 |
| Missing | 151 | 31.2 | 101 | 36.46 | 50 | 24.15 |
| Item 5: In the past 4 weeks, did (S) balance his/her checkbook or a credit card statement? |  |  |  |  |  |  |
| 1=Yes | 211 | 43.6 | 120 | 43.32 | 91 | 43.96 |
| 2=No | 256 | 52.89 | 145 | 52.35 | 111 | 53.62 |
| 3=I don’t know | 10 | 2.07 | 7 | 2.53 | 3 | 1.45 |
| Missing | 7 | 1.45 | 5 | 1.81 | 2 | 0.97 |
| If yes, which best describes how he/she usually performed? |  |  |  |  |  |  |
| 0=With physical help | 9 | 1.86 | 4 | 1.44 | 5 | 2.42 |
| 1=With supervision | 21 | 4.34 | 15 | 5.42 | 6 | 2.9 |
| 2=Without supervision or help | 181 | 37.4 | 101 | 36.46 | 80 | 38.65 |
| Missing | 273 | 56.4 | 157 | 56.68 | 116 | 56.04 |
| Item 6: In the past 4 weeks, did (S) ever write things down? |  |  |  |  |  |  |
| 1=Yes | 445 | 91.94 | 251 | 90.61 | 194 | 93.72 |
| 2=No | 30 | 6.2 | 19 | 6.86 | 11 | 5.31 |
| 3=I don’t know | 2 | 0.41 | 2 | 0.72 | 0 | 0 |
| Missing | 7 | 1.45 | 5 | 1.81 | 2 | 0.97 |
| If yes, which best describes the most complicated things that he/she wrote? |  |  |  |  |  |  |
| 0=His/her signature or name | 45 | 9.3 | 22 | 7.94 | 23 | 11.11 |
| 1=Short notes or messages that other people understood | 227 | 46.9 | 136 | 49.1 | 91 | 43.96 |
| 2=Letters or long notes that other people understood | 173 | 35.74 | 93 | 33.57 | 80 | 38.65 |
| Missing | 39 | 8.06 | 26 | 9.39 | 13 | 6.28 |
| Item 7: In the past 4 weeks, did (S) clean a load of laundry? |  |  |  |  |  |  |
| 1=Yes | 280 | 57.85 | 109 | 39.35 | 171 | 82.61 |
| 2=No | 194 | 40.08 | 161 | 58.12 | 33 | 15.94 |
| 3=I don’t know | 3 | 0.62 | 2 | 0.72 | 1 | 0.48 |
| Missing | 7 | 1.45 | 5 | 1.81 | 2 | 0.97 |
| If yes, which best describes how he/she usually performed: |  |  |  |  |  |  |
| 0=With physical help | 2 | 0.41 | 0 | 0 | 2 | 0.97 |
| 1=With supervision | 16 | 3.31 | 12 | 4.33 | 4 | 1.93 |
| 2=Without supervision or help | 262 | 54.13 | 97 | 35.02 | 165 | 79.71 |
| Missing | 204 | 42.15 | 168 | 60.65 | 36 | 17.39 |
| Item 8: In the past 4 weeks, did (S) keep appointments or meetings with other people, such as relatives, a doctor, the hairdresser, etc.? |  |  |  |  |  |  |
| 1=Yes | 455 | 94.01 | 261 | 94.22 | 194 | 93.72 |
| 2=No | 22 | 4.55 | 11 | 3.97 | 11 | 5.31 |
| 3=I don’t know | 0 | 0 | 0 | 0 | 0 | 0 |
| Missing | 7 | 1.45 | 5 | 1.81 | 2 | 0.97 |
| If yes, which best describes his/her awareness of the meeting ahead of time: |  |  |  |  |  |  |
| 0=Usually did not remember, in spite of verbal reminders on the day | 56 | 11.57 | 31 | 11.19 | 25 | 12.08 |
| 1=Usually remembered the appointment after verbal reminders on the day | 102 | 21.07 | 63 | 22.74 | 39 | 18.84 |
| 2=Usually referred to notes, a diary, or calendar | 260 | 53.72 | 146 | 52.71 | 114 | 55.07 |
| 3=Usually remembered without written or verbal reminders | 37 | 7.64 | 21 | 7.58 | 16 | 7.73 |
| Missing | 29 | 5.99 | 16 | 5.78 | 13 | 6.28 |
| Item 9: In the past 4 weeks, did (S) use a telephone? |  |  |  |  |  |  |
| 1=Yes | 466 | 96.28 | 264 | 95.31 | 202 | 97.58 |
| 2=No | 10 | 2.07 | 7 | 2.53 | 3 | 1.45 |
| 3=I don’t know | 1 | 0.21 | 1 | 0.36 | 0 | 0 |
| Missing | 7 | 1.45 | 5 | 1.81 | 2 | 0.97 |
| If yes, which best describes his/her highest level of performance: |  |  |  |  |  |  |
| 0=Did not answer the phone, but spoke when put on the line | 9 | 1.86 | 4 | 1.44 | 5 | 2.42 |
| 1=Answered the phone and spoke to callers; did not make calls | 58 | 11.98 | 36 | 13 | 22 | 10.63 |
| 2=Made calls only to well-known numbers, by using a directory or list | 76 | 15.7 | 40 | 14.44 | 36 | 17.39 |
| 3=Made calls only to well-known numbers, without referring to a directory or list | 41 | 8.47 | 19 | 6.86 | 22 | 10.63 |
| 4=Made any call necessary e.g., after looking up numbers in white or yellow pages, or by dialing directory assistance | 282 | 58.26 | 165 | 59.57 | 117 | 56.52 |
| Missing | 18 | 3.72 | 13 | 4.69 | 5 | 2.42 |
| Item 10: In the past 4 weeks, did (S) make him/herself a meal or snack at home? |  |  |  |  |  |  |
| 1=Yes | 438 | 90.5 | 250 | 90.25 | 188 | 90.82 |
| 2=No | 38 | 7.85 | 21 | 7.58 | 17 | 8.21 |
| 3=I don’t know | 1 | 0.21 | 1 | 0.36 | 0 | 0 |
| Missing | 7 | 1.45 | 5 | 1.81 | 2 | 0.97 |
| If yes, which best describes his/her highest level of food preparation: |  |  |  |  |  |  |
| 0=Obtained food on his/her own, without mixing or cooking it | 32 | 6.61 | 24 | 8.66 | 8 | 3.86 |
| 1=Mixed or combined food items for a meal or snack, without cooking or microwaving (e.g. made a sandwich) | 56 | 11.57 | 36 | 13 | 20 | 9.66 |
| 2=Cooked or microwaved food, with extensive help | 21 | 4.34 | 10 | 3.61 | 11 | 5.31 |
| 3=Cooked or microwaved food, with little or no help | 329 | 67.98 | 180 | 64.98 | 149 | 71.98 |
| Missing | 46 | 9.5 | 27 | 9.75 | 19 | 9.18 |
| Item 11: In the past 4 weeks, did (S) get around (or travel) outside of his/her home? |  |  |  |  |  |  |
| 1=Yes | 465 | 96.07 | 268 | 96.75 | 197 | 95.17 |
| 2=No | 12 | 2.48 | 4 | 1.44 | 8 | 3.86 |
| 3=I don’t know | 0 | 0 | 0 | 0 | 0 | 0 |
| Missing | 7 | 1.45 | 5 | 1.81 | 2 | 0.97 |
| If yes, which best describes his/her optimal performance: |  |  |  |  |  |  |
| 0=Travelled only with physical help, regardless of the trip | 12 | 2.48 | 4 | 1.44 | 8 | 3.86 |
| 1=Travelled only with a chaperone for supervision regardless of the trip | 81 | 16.74 | 35 | 12.64 | 46 | 22.22 |
| 2=Travelled alone, but remained within 1 mile of home | 44 | 9.09 | 28 | 10.11 | 16 | 7.73 |
| 3=Travelled alone, went at least 1 mile away from home | 328 | 67.77 | 201 | 72.56 | 127 | 61.35 |
| Missing | 19 | 3.93 | 9 | 3.25 | 10 | 4.83 |
| Item 12: In the past 4 weeks, did (S) talk about current events? |  |  |  |  |  |  |
| 1=Yes | 433 | 89.46 | 252 | 90.97 | 181 | 87.44 |
| 2=No | 44 | 9.09 | 20 | 7.22 | 24 | 11.59 |
| 3=I don’t know | 0 | 0 | 0 | 0 | 0 | 0 |
| Missing | 7 | 1.45 | 5 | 1.81 | 2 | 0.97 |
| Item 12a: Did (S) talk about regional, national or international events (including sports)? |  |  |  |  |  |  |
| 0=No | 37 | 7.64 | 21 | 7.58 | 16 | 7.73 |
| 1=Yes | 396 | 81.82 | 231 | 83.39 | 165 | 79.71 |
| Missing | 51 | 10.54 | 25 | 9.03 | 26 | 12.56 |
| Item 12b: Did (S) talk about events outside home involving family, friends or neighbors? |  |  |  |  |  |  |
| 0=No | 29 | 5.99 | 21 | 7.58 | 8 | 3.86 |
| 1=Yes | 404 | 83.47 | 231 | 83.39 | 173 | 83.57 |
| Missing | 51 | 10.54 | 25 | 9.03 | 26 | 12.56 |
| Item 12c: Did (S) talk about events that occurred at home that he/she took part in or watched? |  |  |  |  |  |  |
| 0=No | 33 | 6.82 | 24 | 8.66 | 9 | 4.35 |
| 1=Yes | 400 | 82.64 | 228 | 82.31 | 172 | 83.09 |
| Missing | 51 | 10.54 | 25 | 9.03 | 26 | 12.56 |
| Item 12d: Did (S) converse without repeating him/herself, or asking the same questions repeatedly? |  |  |  |  |  |  |
| 0=No | 174 | 35.95 | 93 | 33.57 | 81 | 39.13 |
| 1=Yes | 259 | 53.51 | 159 | 57.4 | 100 | 48.31 |
| Missing | 51 | 10.54 | 25 | 9.03 | 26 | 12.56 |
| Item 13: In the past 4 weeks, did (S) read a magazine, newspaper or book for more than 5 minutes at a time? |  |  |  |  |  |  |
| 1=Yes | 431 | 89.05 | 251 | 90.61 | 180 | 86.96 |
| 2=No | 43 | 8.88 | 20 | 7.22 | 23 | 11.11 |
| 3=I don’t know | 3 | 0.62 | 1 | 0.36 | 2 | 0.97 |
| Missing | 7 | 1.45 | 5 | 1.81 | 2 | 0.97 |
| Item 13a: Did (S) usually select or ask for something to read? |  |  |  |  |  |  |
| 0=No | 11 | 2.27 | 8 | 2.89 | 3 | 1.45 |
| 1=Yes | 420 | 86.78 | 243 | 87.73 | 177 | 85.51 |
| Missing | 53 | 10.95 | 26 | 9.39 | 27 | 13.04 |
| Item 13b: Did (S) usually talk about what he/she read while or shortly after reading (less than 1 hour)? |  |  |  |  |  |  |
| 0=No | 106 | 21.9 | 61 | 22.02 | 45 | 21.74 |
| 1=Yes | 325 | 67.15 | 190 | 68.59 | 135 | 65.22 |
| Missing | 53 | 10.95 | 26 | 9.39 | 27 | 13.04 |
| Item 13c: Did (S) usually talk about what he/she read while 1-24 hours after reading? |  |  |  |  |  |  |
| 0=No | 194 | 40.08 | 116 | 41.88 | 78 | 37.68 |
| 1=Yes | 237 | 48.97 | 135 | 48.74 | 102 | 49.28 |
| Missing | 53 | 10.95 | 26 | 9.39 | 27 | 13.04 |
| Item 14: In the past 4 weeks, did (S) watch television? |  |  |  |  |  |  |
| 1=Yes | 461 | 95.25 | 265 | 95.67 | 196 | 94.69 |
| 2=No | 12 | 2.48 | 5 | 1.81 | 7 | 3.38 |
| 3=I don’t know | 4 | 0.83 | 2 | 0.72 | 2 | 0.97 |
| Missing | 7 | 1.45 | 5 | 1.81 | 2 | 0.97 |
| Item 14a: Did (S) usually select or ask for different programs or his/her favorite show? |  |  |  |  |  |  |
| 0=No | 46 | 9.5 | 21 | 7.58 | 25 | 12.08 |
| 1=Yes | 415 | 85.74 | 244 | 88.09 | 171 | 82.61 |
| Missing | 23 | 4.75 | 12 | 4.33 | 11 | 5.31 |
| Item 14b: Did (S) usually talk about the content of a program while watching it? |  |  |  |  |  |  |
| 0=No | 123 | 25.41 | 72 | 25.99 | 51 | 24.64 |
| 1=Yes | 338 | 69.83 | 193 | 69.68 | 145 | 70.05 |
| Missing | 23 | 4.75 | 12 | 4.33 | 11 | 5.31 |
| Item 14c: Did (S) talk about the content of a program within a day (24 hours) after watching it? |  |  |  |  |  |  |
| 0=No | 232 | 47.93 | 141 | 50.9 | 91 | 43.96 |
| 1=Yes | 229 | 47.31 | 124 | 44.77 | 105 | 50.72 |
| Missing | 23 | 4.75 | 12 | 4.33 | 11 | 5.31 |
| Item 15: In the past 4 weeks, did (S) ever go shopping at a store? |  |  |  |  |  |  |
| 1=Yes | 430 | 88.84 | 247 | 89.17 | 183 | 88.41 |
| 2=No | 47 | 9.71 | 25 | 9.03 | 22 | 10.63 |
| 3=I don’t know | 0 | 0 | 0 | 0 | 0 | 0 |
| Missing | 7 | 1.45 | 5 | 1.81 | 2 | 0.97 |
| Item 15a: Did (S) usually select correct items without supervision or help? |  |  |  |  |  |  |
| 0=No | 59 | 12.19 | 31 | 11.19 | 28 | 13.53 |
| 1=Yes | 371 | 76.65 | 216 | 77.98 | 155 | 74.88 |
| Missing | 54 | 11.16 | 30 | 10.83 | 24 | 11.59 |
| Item 15b: Did (S) usually pay for items on his/her own? |  |  |  |  |  |  |
| 0=No | 62 | 12.81 | 29 | 10.47 | 33 | 15.94 |
| 1=Yes | 368 | 76.03 | 218 | 78.7 | 150 | 72.46 |
| Missing | 54 | 11.16 | 30 | 10.83 | 24 | 11.59 |
| Item 16: In the past 4 weeks, was (S) ever left on his/her own? |  |  |  |  |  |  |
| 1=Yes | 449 | 92.77 | 262 | 94.58 | 187 | 90.34 |
| 2=No | 28 | 5.79 | 10 | 3.61 | 18 | 8.7 |
| 3=I don’t know | 0 | 0 | 0 | 0 | 0 | 0 |
| Missing | 7 | 1.45 | 5 | 1.81 | 2 | 0.97 |
| Item 16a: Was (S) left away from home, for 15 minutes or longer, during the day? |  |  |  |  |  |  |
| 0=No | 48 | 9.92 | 20 | 7.22 | 28 | 13.53 |
| 1=Yes | 401 | 82.85 | 242 | 87.36 | 159 | 76.81 |
| Missing | 35 | 7.23 | 15 | 5.42 | 20 | 9.66 |
| Item 16b: Was (S) left at home, for an hour or longer, during the day? |  |  |  |  |  |  |
| 0=No | 14 | 2.89 | 9 | 3.25 | 5 | 2.42 |
| 1=Yes | 435 | 89.88 | 253 | 91.34 | 182 | 87.92 |
| Missing | 35 | 7.23 | 15 | 5.42 | 20 | 9.66 |
| Item 16c: Was (S) left at home, for less than 1 hour, during the day? |  |  |  |  |  |  |
| 0=No | 7 | 1.45 | 4 | 1.44 | 3 | 1.45 |
| 1=Yes | 442 | 91.32 | 258 | 93.14 | 184 | 88.89 |
| Missing | 35 | 7.23 | 15 | 5.42 | 20 | 9.66 |
| Item 17: In the past 4 weeks, did (S) use a household appliance to do chores? |  |  |  |  |  |  |
| 1=Yes | 447 | 92.36 | 256 | 92.42 | 191 | 92.27 |
| 2=No | 28 | 5.79 | 15 | 5.42 | 13 | 6.28 |
| 3=I don’t know | 2 | 0.41 | 1 | 0.36 | 1 | 0.48 |
| Missing | 7 | 1.45 | 5 | 1.81 | 2 | 0.97 |
| If yes, check all that apply: |  |  |  |  |  |  |
| Washer |  |  |  |  |  |  |
| Box Checked | 272 | 56.2 | 103 | 37.18 | 169 | 81.64 |
| Box Not Checked | 175 | 36.16 | 153 | 55.23 | 22 | 10.63 |
| Missing | 37 | 7.64 | 21 | 7.58 | 16 | 7.73 |
| Dishwasher |  |  |  |  |  |  |
| Box Checked | 239 | 49.38 | 113 | 40.79 | 126 | 60.87 |
| Box Not Checked | 208 | 42.98 | 143 | 51.62 | 65 | 31.4 |
| Missing | 37 | 7.64 | 21 | 7.58 | 16 | 7.73 |
| Range |  |  |  |  |  |  |
| Box Checked | 308 | 63.64 | 149 | 53.79 | 159 | 76.81 |
| Box Not Checked | 139 | 28.72 | 107 | 38.63 | 32 | 15.46 |
| Missing | 37 | 7.64 | 21 | 7.58 | 16 | 7.73 |
| Dryer |  |  |  |  |  |  |
| Box Checked | 268 | 55.37 | 105 | 37.91 | 163 | 78.74 |
| Box Not Checked | 179 | 36.98 | 151 | 54.51 | 28 | 13.53 |
| Missing | 37 | 7.64 | 21 | 7.58 | 16 | 7.73 |
| Power tool(s) |  |  |  |  |  |  |
| Box Checked | 109 | 22.52 | 101 | 36.46 | 8 | 3.86 |
| Box Not Checked | 338 | 69.83 | 155 | 55.96 | 183 | 88.41 |
| Missing | 37 | 7.64 | 21 | 7.58 | 16 | 7.73 |
| Microwave |  |  |  |  |  |  |
| Box Checked | 366 | 75.62 | 209 | 75.45 | 157 | 75.85 |
| Box Not Checked | 81 | 16.74 | 47 | 16.97 | 34 | 16.43 |
| Missing | 37 | 7.64 | 21 | 7.58 | 16 | 7.73 |
| Vacuum |  |  |  |  |  |  |
| Box Checked | 281 | 58.06 | 160 | 57.76 | 121 | 58.45 |
| Box Not Checked | 166 | 34.3 | 96 | 34.66 | 70 | 33.82 |
| Missing | 37 | 7.64 | 21 | 7.58 | 16 | 7.73 |
| Toaster oven |  |  |  |  |  |  |
| Box Checked | 203 | 41.94 | 106 | 38.27 | 97 | 46.86 |
| Box Not Checked | 244 | 50.41 | 150 | 54.15 | 94 | 45.41 |
| Missing | 37 | 7.64 | 21 | 7.58 | 16 | 7.73 |
| Food processor |  |  |  |  |  |  |
| Box Checked | 57 | 11.78 | 15 | 5.42 | 42 | 20.29 |
| Box Not Checked | 390 | 80.58 | 241 | 87 | 149 | 71.98 |
| Missing | 37 | 7.64 | 21 | 7.58 | 16 | 7.73 |
| Other |  |  |  |  |  |  |
| Box Checked | 123 | 25.41 | 77 | 27.8 | 46 | 22.22 |
| Box Not Checked | 324 | 66.94 | 179 | 64.62 | 145 | 70.05 |
| Missing | 37 | 7.64 | 21 | 7.58 | 16 | 7.73 |
| Item 17a: For the 1 or 2 most commonly used appliances, which best describes how (S) usually used them: |  |  |  |  |  |  |
| 1=With physical help | 2 | 0.41 | 1 | 0.36 | 1 | 0.48 |
| 2=With supervision (e.g., instructions), but no physical help | 15 | 3.1 | 11 | 3.97 | 4 | 1.93 |
| 3=Without help, but operated only on/off controls | 39 | 8.06 | 25 | 9.03 | 14 | 6.76 |
| 4=Without help, and operated all necessary controls | 391 | 80.79 | 219 | 79.06 | 172 | 83.09 |
| Missing | 37 | 7.64 | 21 | 7.58 | 16 | 7.73 |
| Item 18: In the past 4 weeks, did (S) perform a pastime, hobby or game? |  |  |  |  |  |  |
| 1=Yes | 444 | 91.74 | 259 | 93.5 | 185 | 89.37 |
| 2=No | 32 | 6.61 | 13 | 4.69 | 19 | 9.18 |
| 3=I don't know | 1 | 0.21 | 0 | 0 | 1 | 0.48 |
| Missing | 7 | 1.45 | 5 | 1.81 | 2 | 0.97 |
| If yes, check all that apply: |  |  |  |  |  |  |
| Card or board games |  |  |  |  |  |  |
| Box Checked | 130 | 26.86 | 71 | 25.63 | 59 | 28.5 |
| Box Not Checked | 314 | 64.88 | 188 | 67.87 | 126 | 60.87 |
| Missing | 40 | 8.26 | 18 | 6.5 | 22 | 10.63 |
| Bingo |  |  |  |  |  |  |
| Box Checked | 20 | 4.13 | 7 | 2.53 | 13 | 6.28 |
| Box Not Checked | 424 | 87.6 | 252 | 90.97 | 172 | 83.09 |
| Missing | 40 | 8.26 | 18 | 6.5 | 22 | 10.63 |
| Musical instrument |  |  |  |  |  |  |
| Box Checked | 24 | 4.96 | 12 | 4.33 | 12 | 5.8 |
| Box Not Checked | 420 | 86.78 | 247 | 89.17 | 173 | 83.57 |
| Missing | 40 | 8.26 | 18 | 6.5 | 22 | 10.63 |
| Reading |  |  |  |  |  |  |
| Box Checked | 348 | 71.9 | 200 | 72.2 | 148 | 71.5 |
| Box Not Checked | 96 | 19.83 | 59 | 21.3 | 37 | 17.87 |
| Missing | 40 | 8.26 | 18 | 6.5 | 22 | 10.63 |
| Tennis |  |  |  |  |  |  |
| Box Checked | 20 | 4.13 | 15 | 5.42 | 5 | 2.42 |
| Box Not Checked | 424 | 87.6 | 244 | 88.09 | 180 | 86.96 |
| Missing | 40 | 8.26 | 18 | 6.5 | 22 | 10.63 |
| Crosswords |  |  |  |  |  |  |
| Box Checked | 111 | 22.93 | 51 | 18.41 | 60 | 28.99 |
| Box Not Checked | 333 | 68.8 | 208 | 75.09 | 125 | 60.39 |
| Missing | 40 | 8.26 | 18 | 6.5 | 22 | 10.63 |
| Knitting |  |  |  |  |  |  |
| Box Checked | 13 | 2.69 | 0 | 0 | 13 | 6.28 |
| Box Not Checked | 431 | 89.05 | 259 | 93.5 | 172 | 83.09 |
| Missing | 40 | 8.26 | 18 | 6.5 | 22 | 10.63 |
| Gardening |  |  |  |  |  |  |
| Box Checked | 174 | 35.95 | 99 | 35.74 | 75 | 36.23 |
| Box Not Checked | 270 | 55.79 | 160 | 57.76 | 110 | 53.14 |
| Missing | 40 | 8.26 | 18 | 6.5 | 22 | 10.63 |
| Workshop |  |  |  |  |  |  |
| Box Checked | 49 | 10.12 | 46 | 16.61 | 3 | 1.45 |
| Box Not Checked | 395 | 81.61 | 213 | 76.9 | 182 | 87.92 |
| Missing | 40 | 8.26 | 18 | 6.5 | 22 | 10.63 |
| Art |  |  |  |  |  |  |
| Box Checked | 20 | 4.13 | 6 | 2.17 | 14 | 6.76 |
| Box Not Checked | 424 | 87.6 | 253 | 91.34 | 171 | 82.61 |
| Missing | 40 | 8.26 | 18 | 6.5 | 22 | 10.63 |
| Sewing |  |  |  |  |  |  |
| Box Checked | 37 | 7.64 | 2 | 0.72 | 35 | 16.91 |
| Box Not Checked | 407 | 84.09 | 257 | 92.78 | 150 | 72.46 |
| Missing | 40 | 8.26 | 18 | 6.5 | 22 | 10.63 |
| Golf |  |  |  |  |  |  |
| Box Checked | 40 | 8.26 | 35 | 12.64 | 5 | 2.42 |
| Box Not Checked | 404 | 83.47 | 224 | 80.87 | 180 | 86.96 |
| Missing | 40 | 8.26 | 18 | 6.5 | 22 | 10.63 |
| Fishing |  |  |  |  |  |  |
| Box Checked | 9 | 1.86 | 7 | 2.53 | 2 | 0.97 |
| Box Not Checked | 435 | 89.88 | 252 | 90.97 | 183 | 88.41 |
| Missing | 40 | 8.26 | 18 | 6.5 | 22 | 10.63 |
| Other |  |  |  |  |  |  |
| Box Checked | 174 | 35.95 | 113 | 40.79 | 61 | 29.47 |
| Box Not Checked | 270 | 55.79 | 146 | 52.71 | 124 | 59.9 |
| Missing | 40 | 8.26 | 18 | 6.5 | 22 | 10.63 |
| Item 18a: Did (S) require supervision, or help, to perform any of these hobbies? |  |  |  |  |  |  |
| 1=Help | 6 | 1.24 | 2 | 0.72 | 4 | 1.93 |
| 2=Supervision | 29 | 5.99 | 22 | 7.94 | 7 | 3.38 |
| 3=No supervision required | 409 | 84.5 | 235 | 84.84 | 174 | 84.06 |
| Missing | 40 | 8.26 | 18 | 6.5 | 22 | 10.63 |
